# Physiological and transcriptomic comparisons shed light on the high-temperature stress response mechanisms of *Oncidium* cultivars

**DOI:** 10.1186/s12870-025-07254-7

**Published:** 2025-09-30

**Authors:** Xiaoyan Luo, Mingzhong Huang, Yuanhua Luo, Shuang Shuang Yi, Xiaoyun Yu, Junmei Yin, Chonghui Li, Yi Liao, Shunjiao Lu

**Affiliations:** 1 Tropical Crops Genetic Resources Institute, Chinese Academy of Tropical Agricultural Sciences; Key Laboratory of Crop Gene Resources and Germplasm Enhancement in Southern China, Ministry of Agriculture; The Engineering Technology Research Center of Tropical Ornamental Plant Germplasm Innovation and Utilization; Key Laboratory of Tropical Crops Germplasm Resources Genetic Improvement and Innovation of Hainan Province, Haikou, 571101 China; 2 National Key Laboratory for Tropical Crop Breeding, Sanya Research Institute, Chinese Academy of Tropical Agricultural Sciences, Sanya, 571101 China; 3 Institute of Crop Sciences, Fujian Academy of Agricultural Sciences (Fujian Germplasm Resources Center), Fuzhou, 350013 Fujian China

**Keywords:** *Oncidium*, HT stress, Thermotolerance, Transcriptome, HSPs, Metabolic reprogramming, WGCNA

## Abstract

**Background:**

High-temperature (HT) stress poses a significant threat to plant growth and productivity, necessitating a deeper understanding of thermotolerance mechanisms in economically important species like *Oncidium* orchids. This study investigates the physiological and molecular responses of heat-tolerant (GR) and heat-sensitive (HC) *Oncidium* cultivars under HT stress to identify key adaptive strategies.

**Results:**

Physiological analyses revealed that GR maintained superior chlorophyll retention, membrane stability, and metabolic flexibility under HT stress, while HC exhibited severe photosynthetic collapse and oxidative damage. Transcriptomic profiling identified 26,683 differentially expressed genes (DEGs) in GR, with pronounced upregulation of heat shock proteins (*HSP20*, *HSP70*, *HSP90*), antioxidant enzymes (glutathione peroxidase), and chloroplast-stabilizing genes. Functional enrichment analyses highlighted GR’s coordinated activation of protein homeostasis (GO:0044267), photosynthetic protection (GO:0009522), and metabolic reprogramming (ko01100), including glutathione metabolism (ko00480) and phenylpropanoid biosynthesis (ko00940). Weighted gene co-expression network analysis (WGCNA) further underscored GR’s robust transcriptional network, dominated by heat-shock proteins (HSPs) and heat stress transcription factors (HSFs), whereas HC displayed fragmented stress responses.

**Conclusions:**

Collectively, these results demonstrate that the thermotolerant GR cultivar employs a multi-layered defense strategy, including: (1) predominant upregulation of small heat shock proteins (*HSP20*) rather than canonical HSP70/90; (2) chloroplast protection via oxygen-evolving enhancer proteins; and (3) a well-coordinated gene regulatory network centered on *HSFA2*. Notably, thylakoid membrane stability emerged as an orchid-specific thermotolerance trait. Comparative analysis demonstrated that GR’s multi-layered defense strategy contrasts sharply with HC’s fragmented responses, characterized by protein homeostasis collapse and oxidative damage. Our findings provide both fundamental insights into orchid stress physiology and practical targets (*HSP20*, chloroplast *HSP70*, phenylpropanoid biosynthesis) for developing climate-resilient orchids through molecular breeding approaches.

**Supplementary Information:**

The online version contains supplementary material available at 10.1186/s12870-025-07254-7.

## Background

Global climate change has intensified the frequency and severity of HT stress events, posing a critical threat to plant survival and agricultural productivity [1]. Elevated temperatures disrupt cellular homeostasis by denaturing proteins, damaging membranes, and generating reactive oxygen species (ROS), which collectively impair plant growth and development [2]. To mitigate these effects, plants employ multifaceted defense mechanisms, including the induction of HSPs for protein stabilization [3], activation of antioxidant systems to scavenge ROS [4], accumulation of osmoprotectants [5], and metabolic reprogramming to maintain energy balance [6]. These responses are coordinately regulated by HSFs and other signaling components [7]. While these mechanisms are well-characterized in model plants like Arabidopsis thaliana [8] and staple crops such as rice and wheat [9, 10], ornamental species-particularly epiphytic orchids-remain understudied despite their economic significance [11].

*Oncidium* orchids, renowned for their vibrant floral displays, are commercially vital to the global floriculture industry [12]. Native to tropical and subtropical regions, many *Oncidium* hybrids exhibit limited thermotolerance under cultivation conditions [13]. Research on orchid thermotolerance has progressed significantly through key chronological discoveries. The foundation was laid by Chin et al. [14], who first demonstrated that prolonged heat exposure induces floral transition in *Oncidium* hybrids through upregulation of cytosolic ascorbate peroxidase 1 (*APX1*), establishing its crucial role in redox homeostasis regulation. Building on this, Chin et al. [15] subsequently revealed that overexpression of *Oncidium CytAPX1* in Arabidopsis enhanced abiotic stress tolerance by modulating ROS scavenging. Parallel investigations in related orchid species by Fan et al. [16] uncovered that exogenous calcium application boosts heat tolerance in *Dendrobium* nobile through modulation of HSPs (*HSPA1s*, *HSP90A*) and transcription factors (*MYB*, *WRKY*, NAC). More recent advances include Liu et al. (2021)'s identification of *OnWRKY1*, a heat- and salicylic acid (SA)-inducible transcription factor in *Oncidium* that implicates SA signaling in thermotolerance responses [17], followed by Kerchev and Van Breusegem’s elucidation of *Oncidium APX*’s dual catalytic activity utilizing both ascorbate and glutathione substrates [18]. However, a comprehensive understanding of the physiological and molecular basis of HT tolerance in *Oncidium*—particularly the divergent responses between heat-tolerant and heat-sensitive cultivars—is still lacking.

Here, we employed an integrative approach combining phenotyping, physiological assays, and transcriptomics to dissect HT adaptation in *Oncidium*. Using a heat-tolerant cultivar ‘Gower Ramsey’, GR) and a heat-sensitive cultivar (‘Hwuluduen Chameleon’, HC), we: (1) quantified key physiological indicators, including chlorophyll retention, membrane stability, and osmoprotectant accumulation; (2) identified transcriptomic signatures associated with GR’s superior thermotolerance; and (3) proposed candidate genes and pathways for targeted breeding of climate-resilient orchids. Our findings not only advance the understanding of *Oncidium*-specific HT adaptation but also contribute to the broader field of plant stress biology by highlighting conserved and unique thermotolerance strategies.

## Methods

### Plant materials

Two *Oncidium* cultivars with contrasting heat tolerance were selected for this study: the heat-sensitive cultivar *Oncidium cv*. HC and the heat-tolerant cultivar *Oncidium cv*. GR. These cultivars were obtained from the orchid resource nursery of the Tropical Flower Research Center, Institute of Tropical Crop Variety Resources, Chinese Academy of Tropical Agricultural Sciences. In our previous study [19], we systematically evaluated the heat tolerance of multiple *Oncidium* cultivars and confirmed that HC and GR exhibit significant differences in thermotolerance, providing a reliable experimental system for further mechanistic investigations.

To ensure genetic uniformity, both cultivars were propagated via tissue culture (clonal propagation). After deflasking, the plantlets were transplanted into sphagnum moss-filled pots (10 cm diameter) and maintained in a greenhouse. Nine-month-old acclimatized plants (post-transplantation) with uniform growth were selected for experiments.

### Treatment conditions and sampling strategy for physiological and transcriptomic analyses in *Oncidium* plants

To precisely characterize the physiological responses of heat-tolerant GR and heat-sensitive HC varieties under high-temperature stress and further confirm their thermotolerance differences, we conducted a series of physiological measurements under varying temperature regimes. Plant materials were transferred from the greenhouse and acclimated for 7 days in growth chambers at a constant 22 °C (day/night) to ensure temperature was the sole variable. The plants were then divided into three treatment groups (35 °C, 40 °C, and 45 °C) with controls maintained at 22 °C. Leaf samples were collected at 0, 2, 4, 8, 12, 24, and 48 h after stress initiation, except for the 45 °C treatment where sampling concluded at 12 h due to complete plant desiccation.

To elucidate the molecular mechanisms underlying the differential heat responses between thermotolerant GR and heat-sensitive HC varieties, we conducted transcriptome sequencing under controlled heat stress conditions. Based on our physiological analyses revealing the most pronounced differential responses at 40 °C, we selected this temperature for transcriptomic profiling to maximize the detection of stress-responsive genes while maintaining sample viability. The sampling strategy incorporated an additional 1 h time point to capture early transcriptional events, with focused analysis of the critical 0–12 h window.

This early-phase sampling strategy was implemented because: (1) heat-sensitive HC plants exhibited visible dehydration symptoms beyond 12 hs, compromising RNA integrity; (2) molecular responses typically precede and trigger subsequent physiological adaptations; and (3) the most dynamic transcriptional reprogramming occurs within the initial 12 hs of stress exposure, capturing the primary regulatory events in heat response pathways[20, 21].

### Measurement of physiological indicators under high-temperature stress

Five key physiological parameters were analyzed: (1) leaf water content (gravimetric determination), (2) relative electrolyte conductivity (REC, modified from Seitz et al.) [22], (3) malondialdehyde (MDA) content, (4) proline (PRO) concentration, (5) soluble sugar levels (anthrone-sulfuric acid method). MDA, PRO, and soluble sugar contents were quantified using commercial assay kits (Solarbio Science & Technology, Beijing), while leaf water content was determined by standard oven-drying method. All measurements followed established protocols with appropriate controls and replicates.

### Transcriptome sequencing and analysis

RNA was extracted using TRIzol® reagent (Thermo Fisher Scientific, Waltham, MA, USA) and treated with RNase-free DNase I (Takara Bio, Kusatsu, Japan). RNA quality and integrity were assessed through multiple approaches: electrophoresis on 1% agarose gels, absorbance ratio measurements (A260/280 and A260/230) using a NanoDrop spectrophotometer (Thermo Scientific, USA), and RNA integrity number (RIN) determination with an Agilent 2100 Bioanalyzer (Agilent Technologies, USA). Only samples with A260/280 ratios between 1.8 and 2.0, A260/230 >2.0, and RIN ≥ 7.0 were used for library construction.

Sequencing libraries were prepared from 1.5 μg of high-quality RNA per sample using the NEBNext® Ultra™ RNA Library Prep Kit (New England Biolabs, Ipswich, MA, USA) following the manufacturer’s protocol. The library preparation workflow included: (1) poly(A)+ mRNA enrichment using oligo-dT beads, (2) fragmentation of mRNA, (3) first- and second-strand cDNA synthesis, (4) end repair and A-tailing, (5) adapter ligation with unique barcodes for each sample, and (6) size selection (200–250 bp) using AMPure XP beads. The final libraries were quantified using a Qubit fluorometer (Thermo Fisher Scientific) and assessed for quality with an Agilent Bioanalyzer 2100 before being sequenced on the Illumina Novaseq 6000 platform (Beijing Allwegene Technology) to generate 150 bp paired-end reads.

For data analysis, raw sequencing reads were first processed to remove adapter sequences, low-quality reads (Q-score < 20), and reads containing poly-N sequences (>10% N bases). The clean reads were then de novo assembled using Trinity (v2.8.5) with default parameters. To improve assembly quality, redundant transcripts were removed using CD-HIT (identity cutoff = 0.95) and Corset for clustering, while BUSCO (v5.2.2) was employed to evaluate the completeness of the transcriptome assembly against the eukaryota_odb10 database. Functional annotation was performed by searching against seven public databases: *NCBI non-redundant protein* (NR), *NCBI nucleotide* (NT), KEGG Orthology (KO), Swiss-Prot, Pfam, KOG, and Gene Ontology (GO) using BLAST (e-value ≤ 1e−5).

Gene expression levels were quantified using RSEM (v1.3.3) and normalized as FPKM (Fragments Per Kilobase of transcript per Million mapped reads). Differential expression analysis between experimental groups was performed using DESeq2 (v1.10.1) with the following stringent criteria: (1) adjusted P-value (padj) < 0.05 after Benjamini-Hochberg correction for multiple testing, and (2) absolute log2 fold change (|log2FC|) >1. The resulting DEGs were further analyzed for functional enrichment using GOseq (v1.34.1) for GO terms and KOBAS (v3.0) for KEGG pathways, with statistical significance set at padj < 0.05. Additionally, k-means clustering was applied to identify co-expressed gene patterns under different experimental conditions.

### Validation of RNA-seq by quantitative real-time PCR (qRT-PCR)

To validate the RNA-seq results, we selected 12 representative DEGs for qRT-PCR analysis with *OnActin* as the reference gene (primers listed in Table S1). First-strand cDNA was synthesized from 1 μg total RNA using the PrimeScript RT reagent kit (Takara Bio, Japan). Three biological and technical replicates were performed per gene. Relative expression levels were calculated via the 2-^ΔΔ^Ct method and showed significant correlation with RNA-seq data (Pearson’s r >0.85, *p* < 0.01), confirming transcriptome reliability.

### Statistical analysis

Data were analyzed using SPSS 17.0 (IBM, USA) and R 4.5.0 (R Foundation). For physiological data, one-way ANOVA assessed temperature (35 °C, 40 °C, 45 °C) and time (0–48 h) effects within each cultivar (GR, HC), with post-hoc Duncan’s test (*p* < 0.05). Three-way ANOVA evaluated cultivar × temperature × time interactions. Transcriptomic DEGs were identified by DESeq2 (|log_2_^FC^| >1, *padj* < 0.05), and WGCNA linked gene modules to traits. Functional enrichment (GOseq/KOBAS, *padj* < 0.05) and PCA validated reproducibility across biological triplicates.

## Results

### Phenotypic differences between thermotolerant and heat-sensitive cultivars under HT stress

**Fig. 1:**
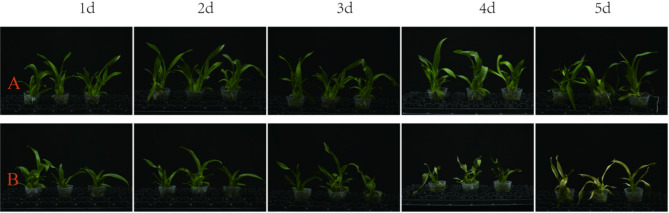
Phenotypes of two *Oncidium* varieties under HT stress (42 °C/38 °C, Day/Night,) for five consecutive days in previous experiments. **A** Heat-resistant cultivar ‘*Oncidium.*cv GR. **B** Heat-sensitive cultivar’*Oncidium.*cv HC

To validate our preliminary evaluation results, we conducted simulated natural high-temperature stress experiments on both GR and HC varieties. The results demonstrated that, under controlled high-temperature stress conditions (42 °C day/38°C night), marked differences in phenotypic responses (Fig. 1) and physiological performance (Table 1) were observed between the thermotolerant (GR) and heat-sensitive (HC) cultivars of *Oncidium* orchids. Chlorophyll content quantification revealed significant cultivar-specific responses to thermal stress.

**Table 1:** Changes in chlorophyll content (mg/g^FW^) in thermotolerant (GR) and heat-sensitive (HC) *Oncidium* cultivars under high-temperature stress

Variety	CK	1d	2d	3d	4d	5d
GR	5.31±0.18	4.05±0.46	4.84±0.41	4.05±0.46	3.70±0.12	2.39±0.19
HC	5.26±0.11	3.66±0.94	2.74±0.12	2.48±0.13	1.87±0.05	0.17±0.04

Data represent mean ± SE (n = 3). CK: control conditions; 1d–5d: days under high-temperature stress (42 °C day/38 °C night) (*p* < 0.05 by two-way ANOVA with Tukey’s post-hoc test)

HC cultivar exhibited rapid physiological deterioration, with chlorophyll content declining sharply from 5.27 ± 0.11 mg/g^FW^ at control conditions to 0.17 ± 0.05 mg/g^FW^ by day 5 (*p* < 0.01). This 96.8% reduction correlated with visible stress symptoms appearing as early as day 2, including leaf turgor loss and wilting. By day 3, when chlorophyll levels had decreased to 2.48 ± 0.13 mg/g^FW^ (52.9% reduction), leaf tip scorching and necrosis became apparent. The progressive chlorophyll degradation (demonstrated by daily reductions of 1.74-2.98 mg/g^FW^) mirrored the visual symptom progression, culminating in complete leaf desiccation by day 5.

In contrast, the thermotolerant (GR) cultivar maintained significantly higher chlorophyll retention throughout the stress period (*p* < *0.05*). Starting from 5.31 ± 0.18 mg/g^FW^ under control conditions, GR plants retained 45.1% of initial chlorophyll (2.39 ± 0.19 mg/g^FW^) at day 5. This physiological stability corresponded with delayed symptom developmentno visible stress signs were observed until day 4, when chlorophyll levels first dropped below 4.0 mg/g^FW^. Even at day 5, GR plants showed only mild leaf softening, maintaining substantially higher chlorophyll content than HC plants at all time points (2.4 vs 0.17 mg/g^FW^, *p* < 0.001).

The differential chlorophyll degradation patterns between cultivars (GR showing gradual linear decline vs HC’s exponential decrease) suggest distinct protective mechanisms. The GR cultivar’s ability to maintain chlorophyll levels above 4.0 mg/g^FW^ for the first 3 days (compared to HC’s drop below this threshold by day 1) indicates superior photosystem stability. These quantitative measurements confirm that chlorophyll content serves as a sensitive indicator of heat stress response in *Oncidium*, with the GR cultivar demonstrating significantly greater thermotolerance through both physiological maintenance and delayed symptom onset.

### Physiological responses to HT stress in GR and HC varieties

**Fig. 2:**
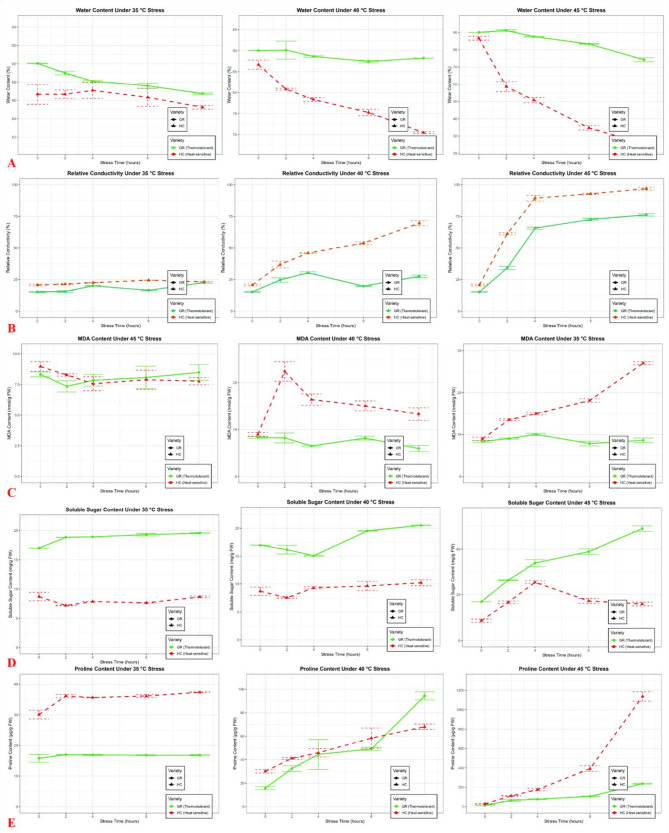
Physiological responses of heat-tolerant (GR) and heat-sensitive (HC) cultivars under different temperature stresses. **A** Water content (%), **B** REC (%), **C** Soluble sugar content (mg/g^FW^), **D** PRO content (μg/g ^FW^), **E** MDA content (nmol/g^FW^). Data show mean ± SD (n = 3) of GR (red) and HC (blue) cultivars exposed to 35 °C (circles), 40 °C (triangles), and 45 °C (squares) over a 48-h period. Asterisks indicate significant differences between cultivars at each time point (*p* < 0.05, *p* < 0.01, *p* < 0.001, two-way ANOVA with Tukey’s post-hoc test)

The physiological responses of two plant varieties, GR (heat-tolerant) and HC (heat-sensitive), to elevated temperatures (35 °C, 40 °C, and 45 °C) were evaluated over a 48-h period (with 45 °C observations limited to 12 hs due to severe stress symptoms). Key parameters—water content, relative conductivity (membrane integrity), PRO (osmoprotectants), soluble sugars (metabolic adjustment), and MDA (oxidative stress marker)—were quantified (Fig. 2A–E).

Under HT stress, the GR and HC varieties exhibited starkly divergent physiological responses. GR demonstrated robust thermotolerance, maintaining stable water content (87–90%) at 35 °C until 48 hs, with gradual declines at higher temperatures (~79% at 40 °C and ~75% at 45 °C) (Fig. 2A). In contrast, HC suffered severe water loss, particularly at ≥40 °C (~58% at 40 °C and ~24% at 45 °C by 48 hs). Membrane integrity followed a similar trend: relative conductivity in HC peaked at ~99% by 12 hs (45 °C), indicating catastrophic damage, while GR maintained lower leakage (~88% at 40 °C) (Fig. 2B).

Osmoprotectant dynamics further highlighted varietal differences. PRO accumulation was extreme in HC (~1,225 μg/g at 45 °C by 12 hs), suggesting acute stress, whereas GR showed later, moderated peaks (~246 μg/g). Soluble sugars remained stable or increased in GR (e.g., ~52 mg/gat 45 °C) but fluctuated erratically in HC (Fig. 2C and D). Oxidative stress (MDA) was severe in HC at 40 °C (~27.70 nmol/g), but lower at 45 °C (~8.43 nmol/g), likely due to tissue collapse; GR maintained manageable MDA levels (<12.51 nmol/g) (Fig. 2E).

Temperature-dependent acclimation strategies were evident: GR exhibited gradual resource mobilization, with sustained water retention and delayed PRO/sugar responses at 35–40 °C, while HC showed rapid physiological failure at ≥40 °C. The 45 °C threshold proved critically lethal for HC but was partially tolerated by GR, underscoring GR’s superior heat adaptation via stabilized membranes and controlled osmoprotectant deployment.

Physiological responses to 40 °C heat stress revealed significant differences between the GR (heat-tolerant) and HC (heat-sensitive) varieties. MDA content, an indicator of oxidative damage, was significantly higher in HC compared to GR across multiple time points (*p* < 0.05, two-tailed t-test), peaking at 27.3 nmol/g at 2 h, while GR maintained levels below 12.5 nmol/g. Membrane integrity, assessed by relative electrolyte leakage, showed severe impairment in HC (> 70% conductivity by 12 h) versus GR (< 50%), demonstrating superior membrane stability in the tolerant variety. Notably, both varieties maintained physiological viability under 40 °C stress, as evidenced by: (1) water content remaining > 55% in HC and > 80% in GR through 48h, (2) absence of MDA values exceeding the 30 nmol/g irreversible damage threshold, and (3) progressive PRO accumulation (HC: 316.4 μg/g; GR: 196.4 μg/g at 48 h) indicating active osmotic adjustment. The sustained metabolic activity coupled with clear genotype-specific responses suggests 40 °C is an appropriate stress intensity for subsequent transcriptomic analysis.

### Transcriptome library construction and DEG analysis in HC and GR under HT stress

**Fig. 3:**
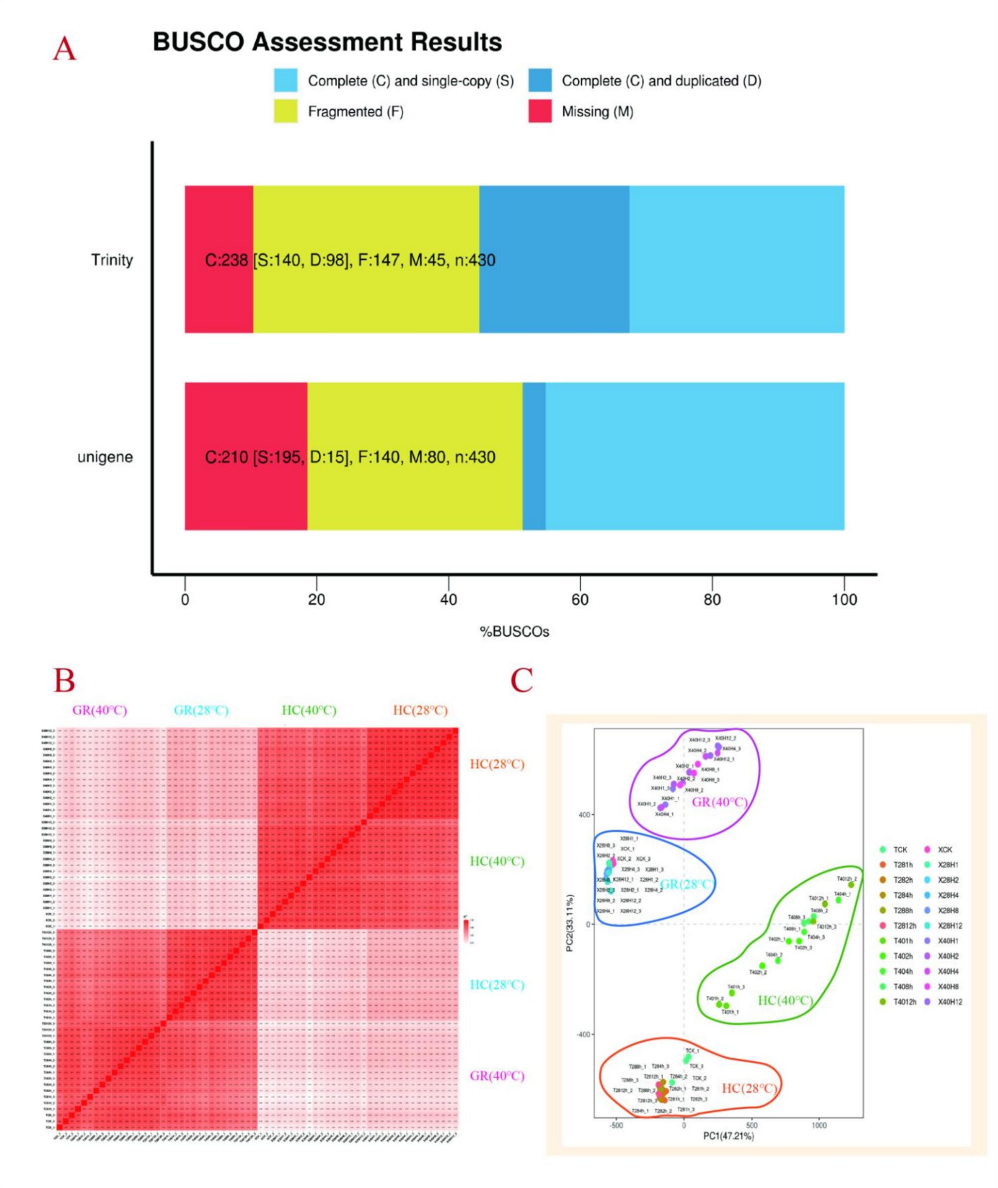
Overview of the illumina transcriptome sequencing. **A** Results of the spliced transcript BUSCO assessment (Different colors represent different spliced transcript types, specifically: C, Complete BUSCOs; S, Complete single-copy BUSCOs; D, Complete duplicated BUSCOs; F, Fragmented BUSCOs; M, Missing BUSCOs; n, Total BUSCO groups searched.) **B** RNA-Seq correlation examination, Heatmap clustering exhibiting the sample correlation analysis of the 22 sequenced samples, clearly segregating into four sample clusters. **C** PCA analysis (Points of varying colors or shapes represent different sample grouping situations, and the scale of the horizontal and vertical axes represents relative distance without practical significance.)

To elucidate the transcriptome profiles of GR and HC in response to HT, the RNA-Seq analyses of GR and HC were performed at 0h, 1 h, 2 h, 4 h, 8 h, and 12 h under 40 °C treatment. A total of 66 RNA-Seq cDNA libraries (Table S2) were constructed, and 37,751,882 to 90,304,386 clean reads (with Q30 percentage ranging from 91.22% to 93.75% and GC content from 44.96 to 48.60%) were obtained (Table S3). The BUSCO assessment results of the assembled transcripts revealed that a rate of 45.3% for complete single-copy BUSCOs, whereas the proportion of complete duplicated BUSCOs accounted for 3.5%. This disparity suggested that the number of genes present as single copies significantly exceeds those found in multiple copies within the genome, implying a satisfactory level of assembly quality (Fig. 3A). Meanwhile, the Pearson’s correlation analysis (Fig. 3B) and principal component analysis (PCA) (Fig. 3C) between 66 samples revealed strong correlation coefficients among biological replications, signifying the reliability and reproducibility of our experimental design and data.

**Fig. 4:**
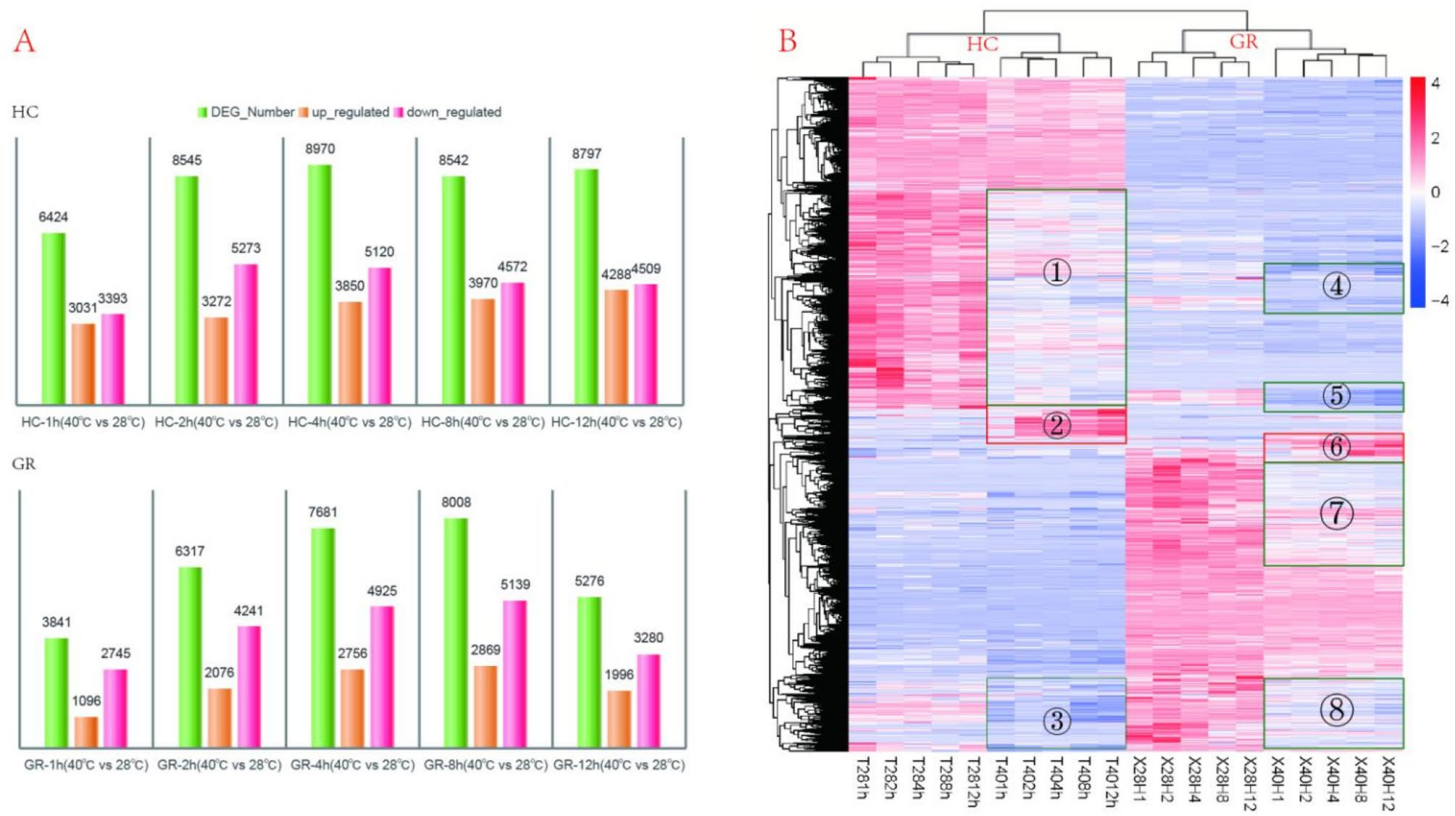
**A** Bar graph depicting the number of DEGs between the stress group (40 °C) and the control group (28 °C) of two varieties at time points 1, 2, 4, 8, and 12 hs under HT stress. **B** The cluster plots of DEGs. *Note* Each column represents a sample (the mean values of the respective triplicate experiments). The colors, ranging from blue to red, indicate the log10 (FPKM+1) values from low to high levels

Then, genes with an FPKM expression level greater than 0.1 were selected for further analysis. A total of 3’841, 6’317, 7’681, 8’008, and 5’276 annotated genes were differentially expressed in the GR at 1, 2, 4, 8, and 12 h of high-temperature treatment, respectively. Similarly, 6’424, 8’545, 8’970, 8’542, and 8’797 annotated genes were differentially expressed in the HC at the same time points (Fig. 4A). Notably, the total number of DEGs in the GR showed an initial increase followed by a decrease, with the total number of DEGs at 12 hs being significantly lower than at 8 hs. In contrast, the total number of DEGs in the HC rapidly increased to 8’545 after 2 hs of treatment and remained above 8’000 until 12 hs, with no declining trend. This suggested that the HT tolerant cultivar GR was able to adapt to the high-temperature stress (40 °C) and gradually adjust to a balanced state. It is worth noting that the number of genes downregulated under HT treatment was greater than that of upregulated genes in both cultivars. Specifically, the number of downregulated genes accounted for 52%, 61%, 57%, 53%, and 51% of the total DEGs in HC at the five time points, respectively. In GR, the corresponding percentages were 71%, 67%, 64%, 64%, and 60%.

The cluster plot of DEGs (Fig. 4B) initially grouped into two categories, representing the HT tolerant cultivar GR and the HT sensitive cultivar HC, respectively. This results indicated that it was significant genetic differences between the two cultivars. Each category was further divided into two subcategories: the normal temperature group and the HT treatment group. Notably, the expression patterns of numerous genes showed significant alterations under HT treatment, including both upregulation and downregulation. However, as observed from the cluster plot, the regions of significantly upregulated and downregulated genes induced by HT in the GR and the HC did not completely overlap. For instance, upregulated genes in HC were mainly concentrated in cluster②, while upregulated genes in GR were mainly concentrated in cluster⑥. The genes represented by these two clusters were largely different, indicated that it was a significant differences in the response mechanisms to high-temperature stress between this two cultivars.

### Functional annotation analysis of DEGs in HC and GR under HT stress

Through transcriptomic analysis of Oncidium under heat stress, we identified 91 classes of significantly upregulated proteins (Table S4), most prominently including heat shock proteins (HSP20, HSP70, HSP90), stress response regulators (HSF_DNA-bind, TCTP), and metabolic mediators (photosynthesis ubiquitin, Pro_CA), while 108 classes were significantly downregulated (Table S5), particularly photosynthesis-related components (RbcS/RuBisCO_small) and metabolic enzymes (GDC-P/Glycine dehydrogenase), revealing distinct molecular signatures of heat response in this orchid species.

### GO enrichment analysis of DEGs in in HC and GR under HT stress

**Fig. 5:**
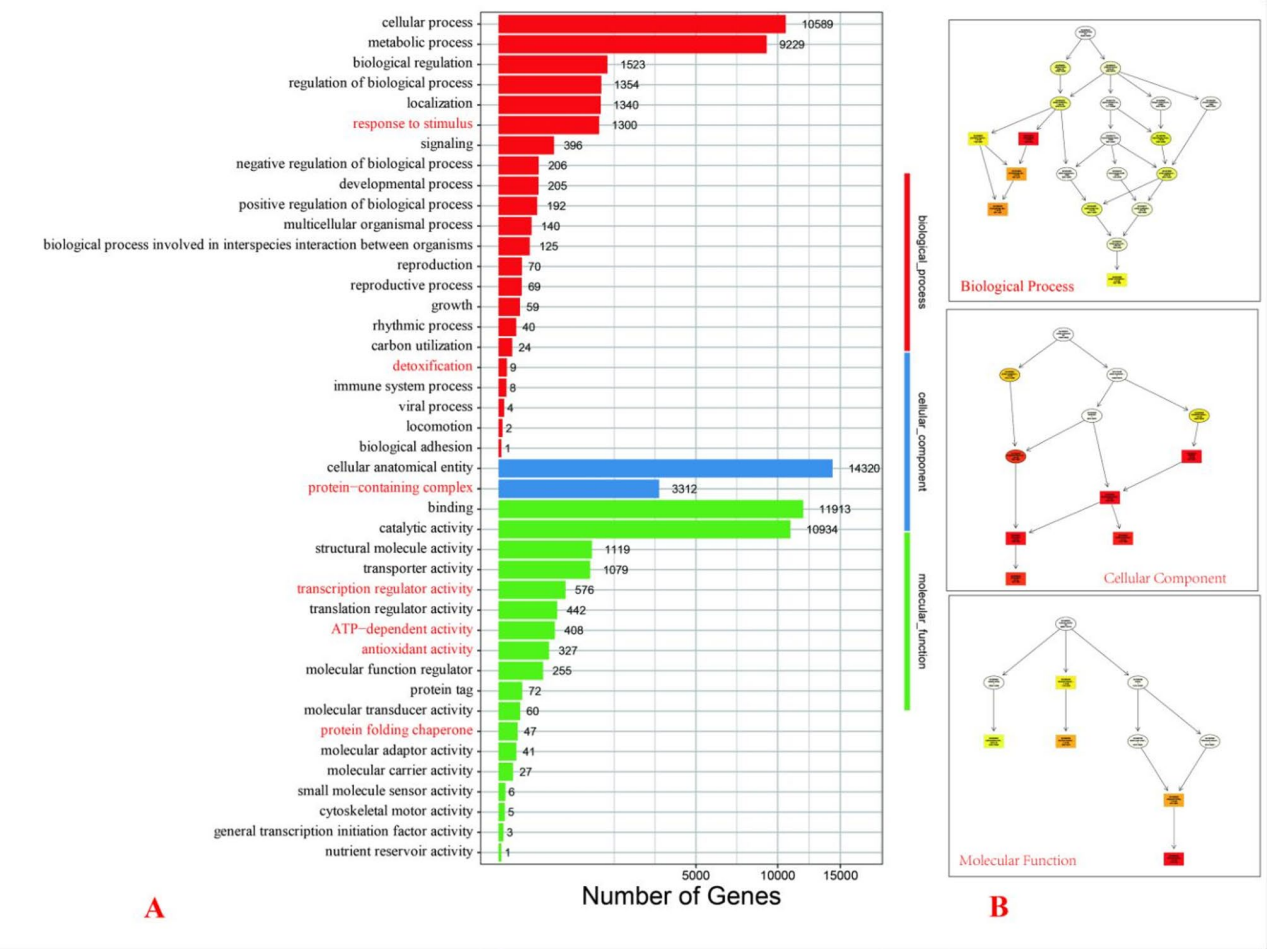
GO Functional Enrichment Analysis of DEGs in GR and HC *Oncidium* under HT Stress. **A** Bar plot: Distribution of DEGs across GO terms (Biological Process, Molecular Function, Cellular Component). Key HT stress-responsive terms (eg. “response to stimulus,” “antioxidant activity,” “protein folding chaperone”) are highlighted in red. **B** TopGO analysis: Significantly enriched terms (*p* < *0.05*) grouped by: biological process: photosynthesis (GO:0015979) and protein metabolism (GO:0019538). Cellular component: chloroplast structures (GO:0009521 [photosystem], GO:0009579 [thylakoid]). Molecular function: oxidoreductase activity (GO:0016491) and chlorophyll binding (GO:0016168)

Our comprehensive GO analysis of heat-sensitive (HC) and heat-tolerant (GR) *Oncidium* varieties under thermal stress reveals distinct molecular adaptation strategies across biological processes, cellular components, and molecular functions (Fig. 5). The highly significant enrichment patterns (*p*<1e^−20^) highlight three fundamental mechanisms underlying GR’s superior thermotolerance.

Firstly, the enrichment analysis revealed critical impacts on the photosynthetic system under high-temperature stress, where photosynthesis-related biological processes (GO:0009765, GO:0015979, GO:0019684; all *p *< 1e^−20^) showed extreme enrichment (1409/4836 DEGs). Notably, at the cellular component level, both photosystem I (GO:0009522, 709/1993 DEGs) and thylakoid membranes (GO:0042651, 1117/3693 DEGs) exhibited severe disruption. Furthermore, molecular function analysis identified dramatic effects on chlorophyll binding (GO:0016168, 474/1412 DEGs) and tetrapyrrole binding (GO:0046906, 831/3805 DEGs). Taken together, these findings strongly suggest that while the heat-tolerant (GR) variety maintains photosynthetic efficiency through specialized protection of pigment-protein complexes, the heat-sensitive (HC) variety suffers from complete photosystem collapse under thermal stress.

Moreover, the analysis demonstrated significant alterations in protein homeostasis maintenance under high-temperature stress. Specifically, cellular protein metabolism (GO:0044267, 2621/14870 DEGs) and modification processes (GO:0006464, 1028/5909 DEGs) showed substantial changes. At the cellular level, protein-containing complexes (GO:0032991, 3312/17389 DEGs) underwent extensive reorganization, while molecular function analysis revealed particularly strong enrichment in structural constituent activity (GO:0003735, 1030/4974 DEGs). These results clearly indicate that the heat-tolerant (GR) variety effectively maintains protein integrity through enhanced folding/chaperone systems and improved stability of structural proteins, thereby providing a crucial adaptive advantage under thermal stress conditions.

Additionally, the data revealed comprehensive metabolic reprogramming in response to high-temperature stress. Most strikingly, the generation of precursor metabolites (GO:0006091, 1150/5054 DEGs, *p *< 1e^−20^) showed strong enrichment, along with dramatic alterations in organonitrogen compound metabolism (GO:1901564, 4305/24892 DEGs). Furthermore, oxidoreductase activity (GO:0016491, 2700/16263 DEGs) demonstrated moderate but significant enrichment (*p *= 5.81e^−15^). These findings collectively suggest that the heat-tolerant (GR) variety strategically adapts to thermal stress by redirecting metabolic flux toward stress-protective compounds while simultaneously maintaining redox homeostasis, which provides a vital survival mechanism under adverse conditions.

Most importantly, the extreme enrichment of thylakoid-related terms across all categories—including biological process, cellular component, and molecular function—strongly suggests that chloroplast membrane stability may serve as the primary determinant of Oncidium heat tolerance. Consequently, these findings not only enhance our understanding of thermotolerance mechanisms in orchids but also provide valuable molecular markers for future thermotolerance breeding programs.

### KEGG pathway enrichment analysis of DEGs in in HC and GR under HT stress

The comparative transcriptomic analysis between the HC and GR varieties of *Oncidium* revealed significant differences in metabolic and signaling pathways, as elucidated by KEGG pathway enrichment. The results highlight distinct molecular mechanisms underlying their differential heat stress responses.

**Fig. 6:**
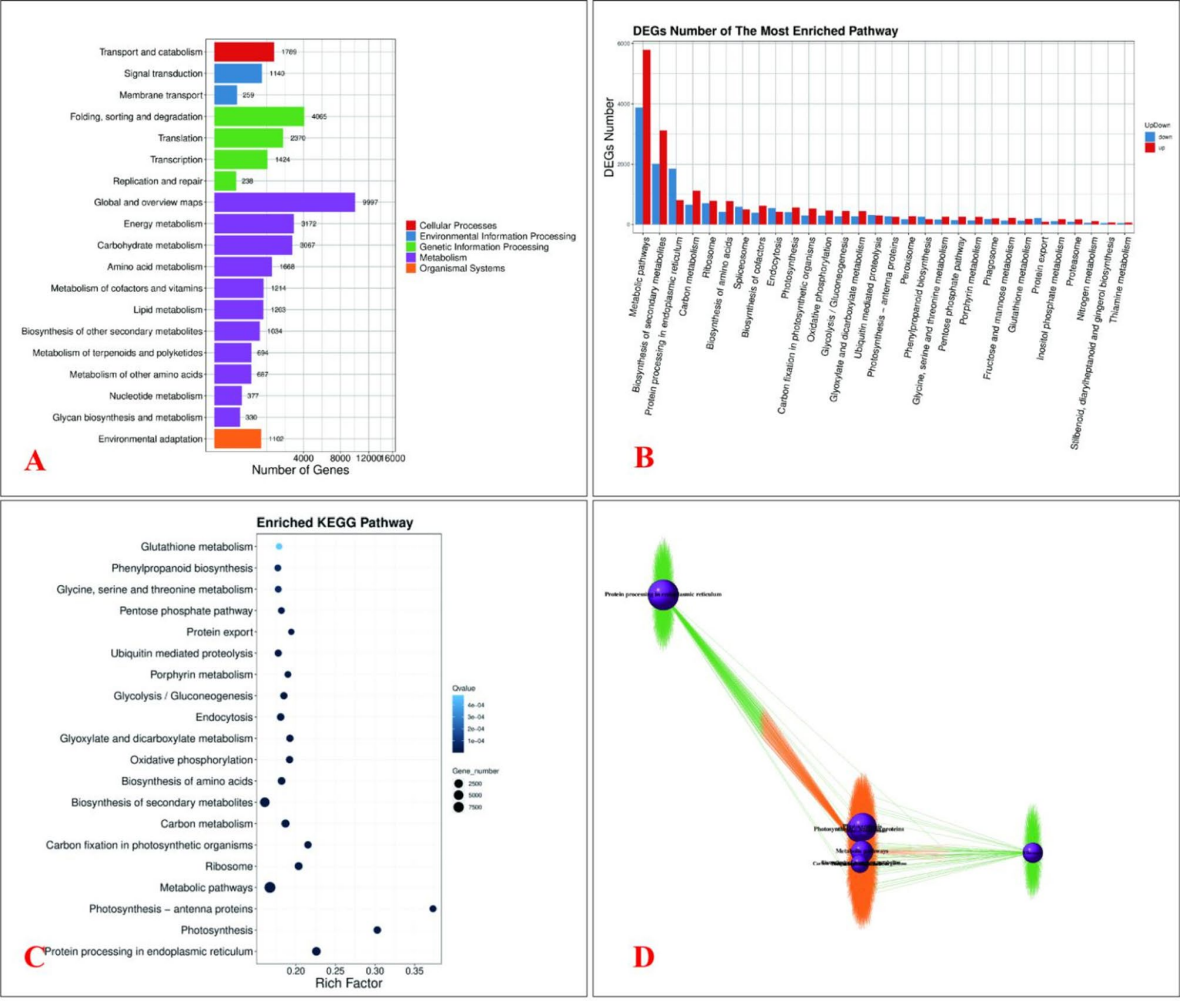
KEGG-based comprehensive analysis of differential gene expression in HC and GR oncidium varieties under HT stress, **A** Functional categorization of KEGG pathways in HC and GR under HT stress, showing gene distribution across five major categories. Key metabolic and stress-responsive pathways are highlighted. **B** Differential expression of KEGG pathways between HC and GR, ranked by DEG abundance. Arrows indicate predominant expression trends in GR (up/down regulation). **C** Enriched KEGG pathways ranked by Rich Factor, with dot size (gene count) and color intensity (significance) highlighting key stress-adaptive pathways in GR. **D** Gene co-expression networks for HC and GR, with node size (connectivity) and color (expression: orange = GR-up, green = HC-up). Labeled clusters reveal temperature-responsive modules

The "Metabolism” category dominated the functional classification, with 9,997 DEGs (the highest among all groups), particularly in subcategories like Global and overview maps (4,065 DEGs), Carbohydrate metabolism (1,424 DEGs), and Amino acid metabolism (1,214 DEGs), suggesting comprehensive metabolic reprogramming in GR. Notably, ”Genetic Information Processing” pathways—including Translation (3,172 DEGs), Transcription (1,668 DEGs), and Folding, sorting, and degradation (1,203 DEGs)—were significantly enriched, highlighting GR’s enhanced capacity for protein homeostasis under stress. In contrast, “Cellular Processes” (e.g., Transport and catabolism; 1,789 DEGs) and “Environmental Information Processing” (e.g., Signal transduction; 259 DEGs) showed moderate but strategic activation, implicating stress signaling and autophagy in thermotolerance. The stark disparity in Energy metabolism (694 DEGs) and Photosynthesis-related pathways (data not shown) further underscores GR’s metabolic efficiency in redistributing resources to sustain viability under heat stress. These results collectively demonstrate that the thermotolerant GR variety prioritizes protein stability, energy flexibility, and stress-responsive metabolism, while HC fails to mount equally robust adaptations, rendering it susceptible to thermal damage. Key pathways like Glutathione metabolism (antioxidant defense) and Phenylpropanoid biosynthesis (cell wall reinforcement) were uniquely upregulated in GR (Fig. 6A), providing molecular targets for future thermotolerance engineering.

Figure 6B shows that the GR exhibited extensive metabolic reprogramming, with the “Metabolic pathways” category (ko01100) showing the highest DEG count (4000 genes), including significant upregulation in Biosynthesis of amino acids (3000 DEGs) and Carbon metabolism (2000 DEGs), suggesting enhanced precursor supply for stress resilience. Critical protein homeostasis pathways were activated in GR, notably Protein processing in endoplasmic reticulum (ko04141; 2000 DEGs) and Ubiquitin-mediated proteolysis (ko04120; 1000 DEGs), reflecting robust chaperone-assisted folding and targeted degradation systems. Stress-responsive pathways like Glutathione metabolism (ko00480; 1000 DEGs) and Phenylpropanoid biosynthesis (ko00940; 1000 DEGs) were uniquely upregulated in GR, correlating with ROS scavenging and structural reinforcement, while energy metabolism shifted from photosynthesis (Photosynthesis-antenna proteins, ko00196; downregulated by 500 DEGs) to glycolysis (Glycolysis/Gluconeogenesis, ko00010; 1000 DEGs). In contrast, the thermosensitive HC variety showed inadequate transcriptional responses, with fewer DEGs across all pathways (e.g., only 200 DEGs in Oxidative phosphorylation versus 1000 in GR). This deficiency was particularly evident in protein homeostasis and antioxidant systems, where HC displayed < 500 DEGs in stress-responsive pathways. The coordinated upregulation of HSPs and downregulation of photosynthetic components in GR likely minimizes photo-oxidative damage, while HC’s limited adaptive capacity explains its thermal vulnerability. These results highlight GR’s multi-layered thermotolerance strategy, integrating metabolic flexibility, protein stability, and redox homeostasis—key targets for future breeding efforts.

The pathway enrichment analysis (Fig. 6C) identified glutathione metabolism (Q = 4e−04; rich factor = 0.35) and phenylpropanoid biosynthesis (Q = 3e−04) as the most significantly enriched pathways in the heat-tolerant GR variety, demonstrating its superior oxidative stress management through ROS scavenging and cell wall reinforcement. GR also exhibited robust activation of protein homeostasis pathways, including ubiquitin-mediated proteolysis (Q = 1e−04) and endoplasmic reticulum protein processing, coupled with metabolic adaptations in glycine/serine/threonine metabolism (Q = 2e−04) and the pentose phosphate pathway—collectively enabling efficient protein turnover, photorespiration, and NADPH generation. Notably, energy metabolism pathways showed divergent regulation: while GR maintained high enrichment of oxidative phosphorylation (Gene_number=2500) and glycolysis/gluconeogenesis, the downregulation of photosynthesis-antenna proteins suggests strategic reallocation of resources from light-dependent reactions to stress-protective metabolism. In contrast, the heat-sensitive HC variety displayed weaker enrichment across all critical pathways, particularly in antioxidative (glutathione metabolism) and proteostatic systems, explaining its susceptibility to thermal damage. The coordinated upregulation of carbon fixation (Gene_number=5000), amino acid biosynthesis, and secondary metabolite production in GR—with 2–3× higher gene counts than HC—underscores its metabolic flexibility to synthesize osmolytes and protective compounds. These results demonstrate that GR’s thermotolerance stems from a multi-layered defense strategy integrating (1) redox homeostasis, (2) protein quality control, and (3) metabolic plasticity, providing specific genetic targets (e.g., glutathione peroxidases, HSP70 chaperones) for improving stress resilience in orchids.

The network visualization (Fig. 6D) revealed distinct thermotolerance mechanisms between the GR and HC varieties. GR exhibited a densely interconnected network with hub genes like HSP70, HSP90, APX, and CAT, which synergistically integrate chaperone activity, antioxidant defense, and metabolic adjustments (e.g., oxidative phosphorylation and glutathione metabolism). Key modules, such as the “double pathways” and “temperature”-associated clusters, highlighted coordinated responses involving heat-shock transcription factors (HSFA2, HSFB1) and sucrose synthases (SUS), underscoring GR’s metabolic flexibility and robust stress adaptation. In contrast, HC’s network was fragmented, with weak coordination between stress signaling (MAPK3) and metabolic genes, alongside disrupted photosynthesis-related clusters (e.g., RBCL, PSBA) and overexpression of degradation-related genes (e.g., ubiquitin ligases). This disorganization likely contributes to HC’s heat susceptibility, as it lacks the specialized modules for secondary metabolite biosynthesis and energy metabolism observed in GR. The systems-level comparison identifies GR’s hub genes and synergistic pathways as key targets for enhancing thermotolerance in orchids.

Collectively, the thermotolerant GR variety exhibits superior heat resilience through a coordinated multi-layered defense strategy, integrating enhanced protein stability (via HSP70/HSP90 chaperones and ubiquitin-mediated proteolysis), robust antioxidant defense (APX/CAT-mediated glutathione metabolism), and metabolic flexibility (shift from photosynthesis to glycolysis/oxidative phosphorylation with upregulated amino acid and phenylpropanoid biosynthesis). In contrast, the thermosensitive HC variety shows fragmented stress responses with deficient protein homeostasis, weak ROS scavenging, and disrupted photosynthesis. Key hub genes (HSFA2, HSP70, APX) and synergistic pathways (glutathione metabolism, protein processing in ER) identified in GR provide critical targets for future breeding.

### Screening of key genes for improving HT tolerance in *Oncidium*

**Fig. 7:**
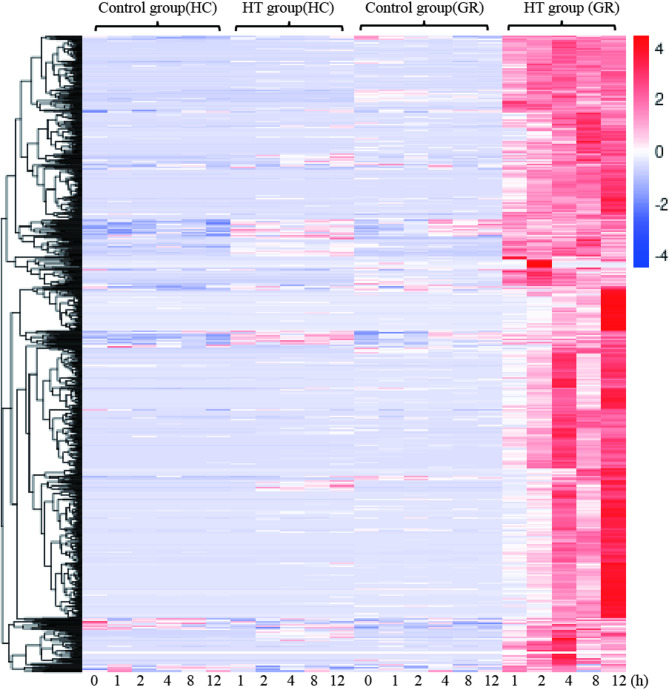
The cluster analysis and thermal map of the 923 unigenes

The genes specifically regulated by HT in the HT-tolerant cultivar GR might play a key role for improving HT tolerance in *Oncidium*. In GR, a total of 26,683 differentially expressed unigenes were obtained (Fig. S1A). The FPKM values of these DEGs at different time points (1 h, 2h, 4h, 8h, 12h) in the two cultivars were retrieved, and k-means cluster analysis was performed based on the FPKM values. Finally, 923 differentially expression unigenes were obtained (Fig. S1B–D), which might be associated with the HT tolerance of *Oncidium*. The cluster analysis and heatmap (Fig. 7) of the 923 unigenes revealed that their expression was up-regulated under HT, and the expression levels were significantly higher in GR than in HC.

The analysis of these unigenes were specifically expressed in GR under HT identified several critical genes linked to heat tolerance in the GR variety of *Oncidium*, including: (1) HSPs: HCRINIHCY_DN76245_c5_g2 (small HSP) and HCRINIHCY_DN80478_c3_g3 (class III HSP) were upregulated in GR, suggesting roles in protein stabilization under thermal stress. (2) Redox Regulators: HCRINIHCY_DN72753_c2_g1 (glutathione peroxidase) and HCRINIHCY_DN77858_c0_g4 (glutathione S-transferase) exhibited enhanced expression in GR, highlighting their importance in mitigating oxidative damage. (3) Metabolic Adjusters: HCRINIHCY_DN89362_c0_g1 (pyruvate kinase) and HCRINIHCY_DN97408_c1_g1 (purple acid phosphatase) were induced in GR, implicating energy metabolism and phosphate homeostasis in heat adaptation. (4) Protein Turnover Genes: HCRINIHCY_DN98194_c1_g2 (E3 ubiquitin ligase) and HCRINIHCY_DN75095_c0_g2 (polyubiquitin) were activated in GR, indicating robust protein degradation and recycling mechanisms. (5) Ribosomal Proteins: HCRINIHCY_DN96792_c0_g1 (mitochondrial 30S ribosomal protein S9) and HCRINIHCY_DN81054_c5_g3 (ribosomal protein L2) showed stable or elevated expression in GR, underscoring translational resilience. Notably, transposon-related genes (e.g., HCRINIHCY_DN76998_c0_g4) were enriched in HC, potentially reflecting genomic instability under stress. These candidate genes collectively suggest that GR’s thermotolerance arises from coordinated activation of chaperones, antioxidant systems, metabolic pathways, and protein homeostasis mechanisms, providing targets for future functional validation and breeding efforts.

### WGCNA analysis of heat-response mechanisms in *Oncidium* varieties

**Fig. 8:**
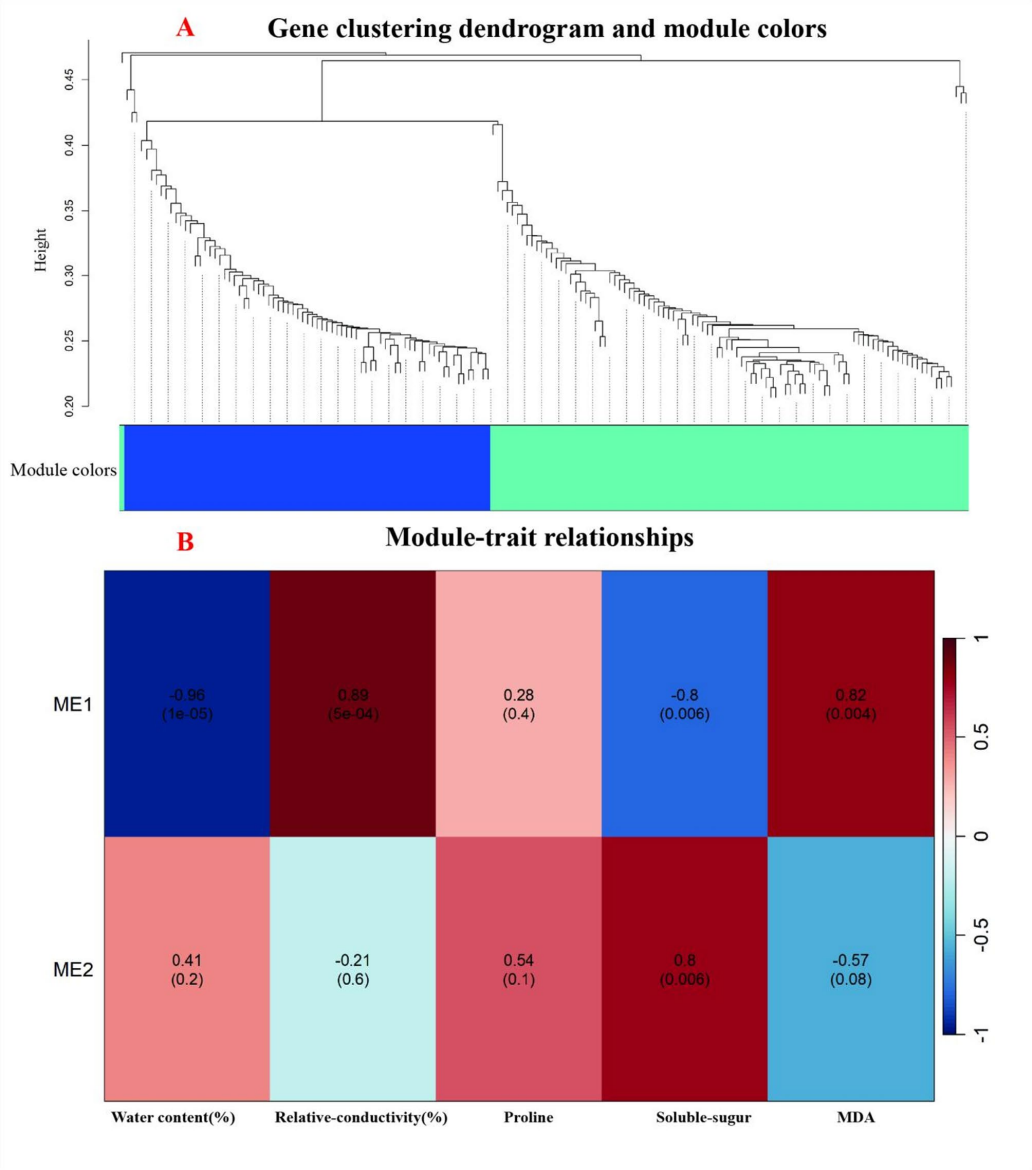
Integrated analysis of gene co-expression networks and module-trait relationships. Panel **A** (Up): gene clustering dendrogram and module colors. The dendrogram illustrates hierarchical clustering of genes based on expression profiles, with the y-axis representing height (dissimilarity measure). Horizontal dashed lines indicate potential cutoffs for defining gene modules at height thresholds (0.25–0.45). Colored bars represent co-expression modules, each assigned a unique color to identify functionally related gene groups. Panel **B** (Down): module-trait relationship heatmap. The heatmap shows correlation coefficients (range: − 1 to 1) and p-values (in parentheses) between gene modules (ME1, ME2, etc.) and measured traits (Wafer content (%), Relative_conductivity (100%), PRO, Subtle_sugur, MDA). Darker shades highlight significant associations (e.g., ME1: Wafer content: r = − 0.96, *p* = 1e−05; Relative_conductivity: r = 0.89, *p* = 5e−04)

Through WGCNA analysis of paired physiological measurements and time-matched transcriptome profiles during heat stress treatment, we identified fundamentally distinct heat-response strategies between heat-tolerant (GR) and heat-sensitive (HC) *Oncidium* varieties (Fig. 8). GR exhibited a well-coordinated defense mechanism, characterized by tight clustering of stress-responsive genes (0.25–0.35 height) in the turquoise module (ME1), which showed strong negative correlations with water retention (− 0.96) and membrane stability (− 0.80). In contrast, HC’s blue module (ME2) displayed disrupted co-expression patterns and positive correlations with stress markers (PRO: 0.89; soluble sugars: 0.82), reflecting its compromised thermotolerance. These findings highlight GR’s evolved ability to maintain cellular homeostasis under heat stress through synchronized gene regulation, while HC’s response appeared disorganized and ineffective. The key thermotolerance genes were identified through their significant upregulation in GR and strong module-trait associations (Table 2).

**Table 2:** Identification and functional analysis of key heat-tolerance related genes in *Oncidium* orchid through co-expression network analysis

Category	Gene ID	Annotation	TPM (GR-12 h)	Module	Function	Correlation
HSPs	TRINITY_DN92890_c1_g5	HSP20 family protein	13,158.65	ME1	Protein stabilization	Water retention: − 0.96
	TRINITY_DN45085_c0_g1	HSP20	151,500.43	ME1	Chaperone activity	Membrane stability: − 0.80
	TRINITY_DN77738_c0_g1	HSP20	4,930.55	ME1	Protein folding	Water retention: − 0.96
	TRINITY_DN103664_c10_g2	HSP20	273,219.91	ME1	Stress response	–
	TRINITY_DN93930_c4_g6	sHSP	136,491.87	ME1	Cytoprotection	–
	TRINITY_DN90635_c3_g1	HSP83-like	–	ME1	Protein refolding	–
	TRINITY_DN96324_c0_g1	HSP70	–	ME1	Protein stabilization	–
	TRINITY_DN83818_c3_g2	Chloroplastic HSP70	–	ME1	Chloroplast protection	–
	TRINITY_DN88803_c0_g1	HSP70	474.49	ME2 (HC)	Oxidative stress response	PRO: + 0.89
	TRINITY_DN84109_c0_g2	HSP70	3,352.51	ME2 (HC)	Compensatory stress response	–
	TRINITY_DN88098_c1_g5	HSP70	1,183.12	ME2 (HC)	Dysregulated folding	–
	TRINITY_DN91885_c0_g1	HSP90	856.22	ME2 (HC)	Stress adaptation	–
Transcription Factors	TRINITY_DN85825_c1_g2	HSF A-2b-like	100-fold Δ	ME1	Heat-responsive gene activation	–
	TRINITY_DN79246_c0_g2	HSF30	1,508.08	ME1	Chaperone regulation	Membrane stability: − 0.80
	TRINITY_DN97742_c3_g1	HSF30-like	458.45	ME1	Stress signaling	–
Photosynthesis-Related	TRINITY_DN88430_c0_g1	Oxygen-evolving enhancer protein	8,404.98	ME1	Photosystem II stability	Water retention: − 0.96
	TRINITY_DN68661_c0_g1	PSBQ-2	–	ME1	Photosynthetic efficiency	–
	TRINITY_DN71295_c0_g1	Chlorophyll a-b binding protein	3,768.10	ME1	Light harvesting	–
	TRINITY_DN68230_c1_g1	Chlorophyll a-b binding protein	–	ME1	Photosystem protection	–
Chaperones & Proteostasis	TRINITY_DN102015_c2_g1	ClpB1 chaperone	1,056.15	ME1	Protein disaggregation	–
	TRINITY_DN67843_c0_g2	ClpB1	851.77	ME1	Protein repair	–
	TRINITY_DN98576_c0_g1	DnaJ homolog	115.23	ME1	Co-chaperone activity	–
	TRINITY_DN77839_c2_g1	Luminal-binding protein BIP1	1,755.30	ME1	ER homeostasis	–
Metabolic Regulators	TRINITY_DN97705_c5_g4	Pyruvate phosphate dikinase	2,766.26	ME1	Carbon metabolism	–
	TRINITY_DN93709_c1_g2	Allantoinase	1,383.81	ME1	Nitrogen recycling	–
	TRINITY_DN103614_c5_g1	Magnesium-protoporphyrin IX cyclase	–	ME1	Chlorophyll biosynthesis	–
Water & Ion Transport	TRINITY_DN86285_c0_g1	Aquaporin	–	ME1	Water channel	Membrane stability: − 0.80
	TRINITY_DN79246_c0_g2	HSF30	1,508.08	ME1	Osmoprotectant regulation	–

The superior thermotolerance of GR was attributed to three key mechanisms: (1) dominance of small HSPs (18/32 upregulated genes, including top candidates TRINITY_DN45085_c0_g1 and TRINITY_DN92890_c1_g5) for rapid protein protection, which showed strong negative correlations with stress markers (*p *< 0.01); (2) maintenance of photosynthetic efficiency through upregulated chloroplast-stabilizing proteins (TRINITY_DN88430_c0_g1 and TRINITY_DN71295_c0_g1); and (3) metabolic flexibility via induced enzymes like pyruvate phosphate dikinase. These adaptive responses, facilitated by GR’s robust scale-free network (R^2 ^= 0.85) dominated by sHSPs and HSFs (TRINITY_DN85825_c1_g2, TRINITY_DN79246_c0_g2), contrast sharply with HC’s reliance on uncoordinated HSP70/90 upregulation (e.g., TRINITY_DN88803_c0_g1) and accumulation of osmotic stress markers (PRO: 0.89; soluble sugars: 0.82). The identified candidate genes - particularly sHSPs, photosynthesis-related proteins, and HSFs - provide valuable targets for molecular breeding of heat-resistant orchid.

### Validation of DEGs by qRT-PCR

**Fig. 9:**
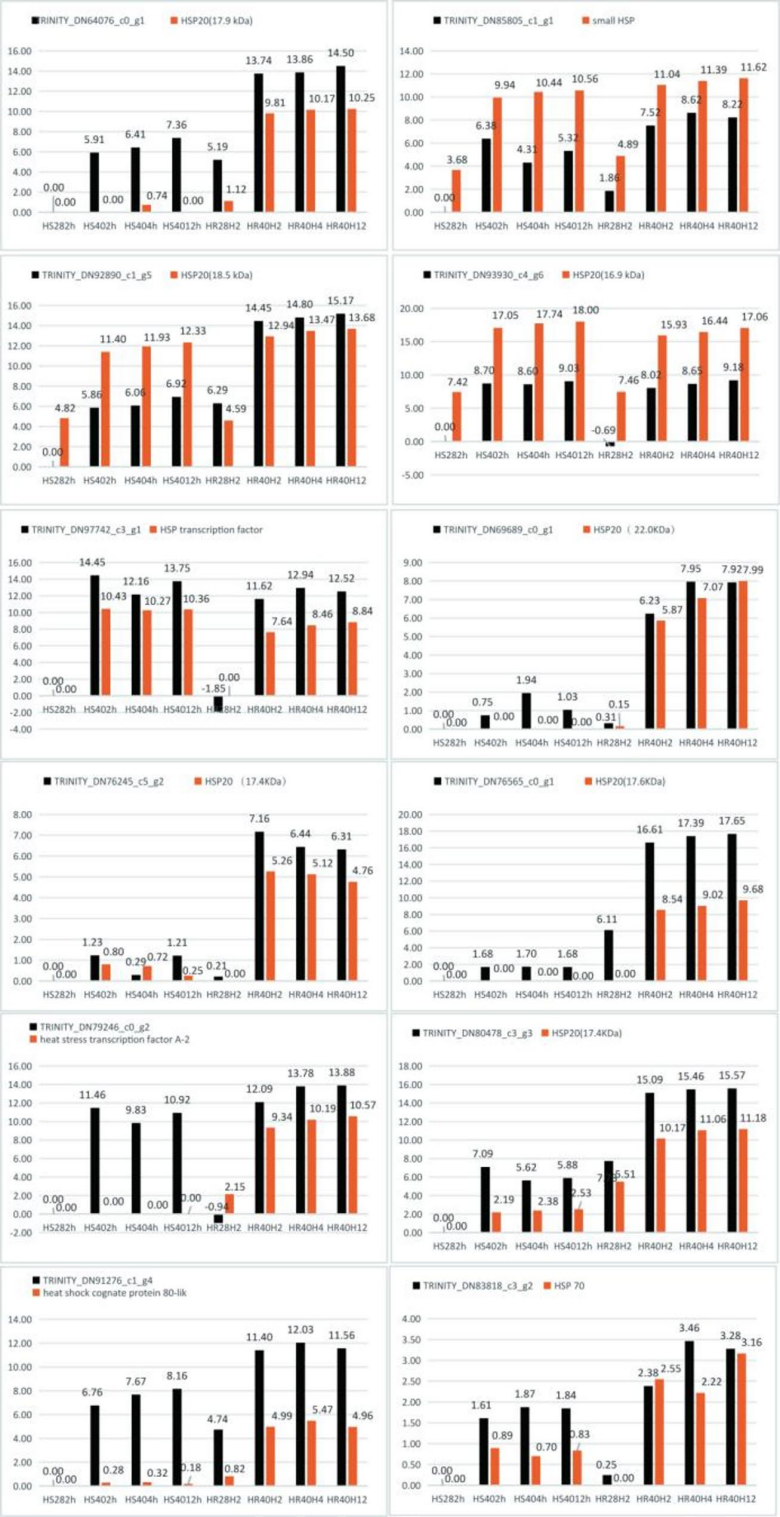
Relative expression levels of 12 DEGs identified in the different comparison groups using RNA sequencing and qRT-PCR. The black bars represent the mRNA expression levels of the twelve DEGs determined by RNA-seq, while the orange bars correspond to the qRT-PCR results for the same DEGs. Log2 (FPKM) was utilized to quantify the expression levels of the genes. The horizontal axes indicate the duration of HT stress (0, 2, 4, and 12 hs, from left to right) in ‘HC’ and ‘GR’, and the vertical axes depict the expression levels

To validate the RNA-seq data, twelve DEGs were selected for real-time PCR analysis. These twelve genes were chosen based on their significant differential expression levels and potential biological relevance. As demonstrated in Fig. 9, the expression patterns of these DEGs were highly consistent between qRT-PCR and RNA-seq data. This strong consistency indicated that the RNA-seq data were accurate and reproducible. Consequently, the RNA-seq data provided a robust foundation for subsequent bioinformatics analyses and the interpretation of the underlying biological processes.

## Discussion

### Phenotypic and physiological mechanisms of HT stress response in *Oncidium*

The thermotolerant GR cultivar of *Oncidium* orchids exhibits a coordinated multi-level defense system against HT stress, as evidenced by three key physiological adaptations. These findings align with established thermotolerance mechanisms in plants while revealing *Oncidium*-specific characteristics.

1. Photosynthetic Stability and Chlorophyll Retention. GR maintained significantly higher chlorophyll content (45.1% retention at day 5 vs. HC’s 96.8% loss) under 42 °C/38 °C stress, correlating with delayed symptom onset. This mirrors findings in other plants where HSPs protect photosynthetic apparatus under HT stress [23]. However, GR’s chlorophyll retention levels (2.39 mg/g^FW^) exceed those reported for most species, suggesting unique adaptations [24]).
2. Membrane Integrity and Osmoprotection. GR showed superior membrane stability (electrolyte leakage: 88% vs. HC’s 99%) and controlled PRO accumulation (246 μg/g vs. HC’s 1225 μg/g). These responses resemble thermotolerant wheat cultivars where membrane stabilization and balanced osmolyte production are critical [25]. HC’s excessive PRO aligns with studies linking hyperaccumulation to cellular damage [26].
3. Metabolic Flexibility. GR maintained stable soluble sugars (52 mg/gat 45 °C) and upregulated glycolysis (1,000 DEGs), while HC showed metabolic disruption. This metabolic reprogramming parallels strategies in heat-adapted Arabidopsis [27]. GR’s coordinated activation of glutathione metabolism (1000 DEGs) further supports its redox homeostasis, a trait less pronounced in HC.

Collectively, GR’s thermotolerance integrates (1) photosynthetic protection, (2) membrane stabilization, and (3) metabolic plasticity—consistent with broad plant thermotolerance paradigms [28, 29] but with *Oncidium*-specific innovations. These findings advance orchid-specific stress physiology understanding.

### Transcriptomic insights into HT stress adaptation in *Oncidium*

The transcriptomic analysis of *Oncidium* under HT revealed distinct molecular mechanisms underlying the thermotolerance of the heat-tolerant cultivar (GR) compared to the heat-sensitive cultivar (HC). These findings align with physiological data, which demonstrated that GR maintained higher membrane stability, photosynthetic efficiency, and water retention under HT stress. At the transcriptomic level, GR exhibited coordinated upregulation of HSPs, antioxidant enzymes, and chloroplast-stabilizing genes, consistent with its superior physiological performance. In contrast, HC showed fragmented stress responses, including dysregulated protein homeostasis and impaired photosynthesis, corroborating its physiological susceptibility to HT.

1. Enhanced protein homeostasis mediated by HSPs and chaperones in GR. Our study identified significant upregulation of HSPs (e.g., HSP20, HSP70, HSP90) and chaperones in GR under HT stress. These proteins play pivotal roles in protein stabilization, folding, and degradation, which are essential for maintaining cellular function under stress. The dominance of small HSPs in GR, particularly their strong negative correlations with stress markers like water retention and membrane stability, underscores their importance in thermotolerance. This aligns with findings in Camellia sinensis, where HSPs were crucial for HT stress adaptation [30]. Similarly, studies in Rhododendron × pulchrum and Capsicum annuum highlighted the role of HSPs in protecting photosynthetic machinery and maintaining protein integrity under HT [31, 32].
2. Superior protection of photosynthetic machinery in GR under HT stres. The GO enrichment analysis revealed severe disruption of photosynthesis-related processes in HC, while GR maintained photosynthetic efficiency through upregulated chloroplast-stabilizing proteins (e.g., oxygen-evolving enhancer proteins and chlorophyll a-b binding proteins). This is consistent with observations in Panicum virgatum, where HT-tolerant varieties exhibited less photosynthetic damage compared to sensitive ones [33]. Additionally, the downregulation of photosynthesis-antenna proteins in GR suggests a strategic reallocation of resources from light-dependent reactions to stress-protective metabolism, a phenomenon also reported in Oryza sativa [34].
3. GR exhibits enhanced metabolic flexibility and oxidative stress management during thermal stress. GR exhibited extensive metabolic reprogramming, including upregulation of glutathione metabolism and phenylpropanoid biosynthesis pathways, which are critical for ROS scavenging and cell wall reinforcement. The enrichment of these pathways in GR correlates with its superior oxidative stress management. Similar metabolic shifts were observed in *Apium graveolens* and *Nicotiana tabacum*, where heat-tolerant varieties activated antioxidant systems and secondary metabolite production to mitigate HT damage [31, 35]. In contrast, HC showed weaker activation of these pathways, leading to oxidative damage, as seen in heat-sensitive Zea mays [36].
4. GR exhibits more integrated transcriptional control during thermal stress. The WGCNA analysis highlighted GR’s well-coordinated gene network, with hub genes like HSFA2 and HSP70 integrating stress responses. This contrasts with HC’s fragmented network, which lacked synergistic regulation. Such coordinated transcriptional responses are consistent with findings in *Dunaliella bardawil*, where thermotolerant strains exhibited tightly regulated stress-responsive modules [37]. The role of HSFs in orchestrating thermotolerance has also been emphasized in Arabidopsis thaliana [38].

Therefore, Our results largely align with existing literature on plant thermotolerance, particularly the conserved roles of HSPs, photosynthetic protection, and metabolic flexibility. However, the unique enrichment of thylakoid-related terms in *Oncidium* suggests chloroplast membrane stability as a key determinant of thermotolerance, a feature less emphasized in other species like *Cajanus cajan* [39]. Additionally, the prominence of small HSPs in GR contrasts with the reliance on HSP70/90 in some crops, indicating species-specific adaptations [40].

### Potential genetic targets for enhancing thermotolerance in *Oncidium*

The transcriptomic and functional analyses of the GR and HC *Oncidium* varieties under HT stress revealed several key genetic targets and molecular mechanisms underlying thermotolerance.

1. HSPs and chaperones. The upregulation of HSP20, HSP70, and HSP90 genes in GR underscores their critical role in protein stabilization and folding under HT stress. The dominance of small HSPs (sHSPs) in GR’s co-expression network aligns with their known function as rapid responders to thermal stress, preventing protein aggregation [41]. Similar findings were reported in wheat and tomato, where sHSPs conferred thermotolerance by maintaining proteostasis [42, 43]. The strong negative correlation between sHSP expression and stress markers (e.g., water retention, membrane stability) in GR further supports their protective role, consistent with studies in Arabidopsis and tobacco [44, 45].
2. Photosystem protection and chloroplast stability. GR exhibited targeted protection of photosynthetic machinery, with upregulated genes like oxygen-evolving enhancer proteins and chlorophyll a-b binding proteins. This contrasts with HC, where photosystem collapse was evident The enrichment of thylakoid-related GO terms (e.g., GO:0009522, GO:0042651) and downregulation of photosynthesis-antenna proteins suggest GR’s strategic reallocation of resources from light-dependent reactions to stress protection. Similar metabolic shifts were observed in thermotolerant wheat and tomato, where chloroplast membrane stability was linked to heat tolerance [46, 47]. The role of HSP70 in chloroplast protection further corroborates findings in tobacco, where chloroplast-targeted HSP70 enhanced thermotolerance [44].
3. Antioxidant defense and redox homeostasis. GR’s activation of glutathione metabolism (ko00480) and upregulation of glutathione peroxidase highlight its superior oxidative stress management. This aligns with studies in wheat and Arabidopsis, where glutathione-related pathways were critical for scavenging ROS under HT [48, 49]. The fragmented antioxidant network in HC mirrors observations in heat-sensitive crops, where inadequate ROS scavenging led to cellular damage [50].
4. Metabolic reprogramming and energy flexibility. GR’s shift from photosynthesis to glycolysis (ko00010) and amino acid biosynthesis reflects metabolic plasticity, a hallmark of thermotolerant species [51]. The induction of pyruvate phosphate dikinase suggests enhanced carbon metabolism, similar to findings in tomato [52]. Conversely, HC’s limited metabolic adjustments (e.g., fewer DEGs in oxidative phosphorylation) align with its susceptibility, as noted in heat-sensitive wheat [42].
5. Protein turnover and ubiquitin-mediated proteolysis. The upregulation of E3 ubiquitin ligases and polyubiquitin genes in GR indicates efficient protein degradation, a mechanism also reported in Arabidopsis for maintaining proteostasis under stress [47]. HC’s overexpression of degradation-related genes (e.g., ubiquitin ligases) without coordinated chaperone activity likely exacerbates protein misfolding, consistent with observations in heat-sensitive rice [53].

## Conclusions

This study demonstrates that the thermotolerant *Oncidium* cultivar GR employs a multi-layered defense strategy against HT stress, characterized by: (1) superior photosynthetic protection through upregulated chloroplast-stabilizing proteins and remarkable chlorophyll retention; (2) enhanced membrane integrity evidenced by reduced electrolyte leakage and balanced osmoprotection; (3) metabolic flexibility via coordinated activation of glycolysis and glutathione metabolism; and (4) transcriptional coordination centered on HSPs (HSP20/70/90), HSFA2, and redox regulators (glutathione peroxidase). Notably, GR’s thylakoid membrane stability emerges as an *Oncidium*-specific thermotolerance trait, while its well-orchestrated gene network contrasts with HC’s fragmented responses. These findings not only identify HSP20, chloroplast-targeted HSP70, and phenylpropanoid biosynthesis as key breeding targets but also provide a framework for developing climate-resilient orchids through future functional validation (e.g., CRISPR of HSFA2) and field trials.

## Supplementary Information

Below is the link to the electronic supplementary material.

Supplementary Material 1.

Supplementary Material 2.

Supplementary Material 3.

Supplementary Material 4.

Supplementary Material 5.

## Data Availability

The transcriptome sequencing data generated in this study are publicly available in the NCBI BioProject database under accession number PRJNA1269304. All supporting data are included in the article and its supplementary materials. The datasets supporting this study are available in public repositories: Raw sequencing data (FASTQ): NCBI SRA [accession number], linked to BioSample accessions SAMN48786736-SAMN48786801. Processed data (DEGs, FPKM): Provided in Supplementary Files [DiffExprAnalysis.zip, fpkm_description.xls]. BioProject metadata: [PRJNA1269304] (https://www.ncbi.nlm.nih.gov/bioproject/PRJNA1269304). Plant materials used are commercially available cultivars (‘Hwuluduen Chameleon’ and ‘Gower Ramsey’).

## Abbreviations

HC: Hwuluduen chameleon
GR: Gower ramsey
REC: Relative electrolyte conductivity
MDA: Malondialdehyde
PRO: Proline
FPKM: Transcripts per million mapped fragments
DEGs: Differentially expressed genes
NR: NCBI non-redundant
NT: NCBI nucleotide
KO: KEGG ortholog
GO: Gene ontology
KEGG: Kyoto encyclopedia of genes and genomes
PCA: Principal component analysis
HSPs: Heat shock proteins
HSFs: Heat stress transcription factors
ROS: Reactive oxygen species
PSII: Photosystem II
