# Supplementary material for: Physiological and transcriptomic comparisons shed light on the high-temperature stress response mechanisms of Oncidium cultivars
Source: BMC Plant Biol. 2025 Sep 30;25:1242. doi: 10.1186/s12870-025-07254-7 (PMC12487527; doi:10.1186/s12870-025-07254-7)
Supplement: Supplementary file 1 — Supplementary Material 1. [file 12870_2025_7254_MOESM1_ESM.docx]

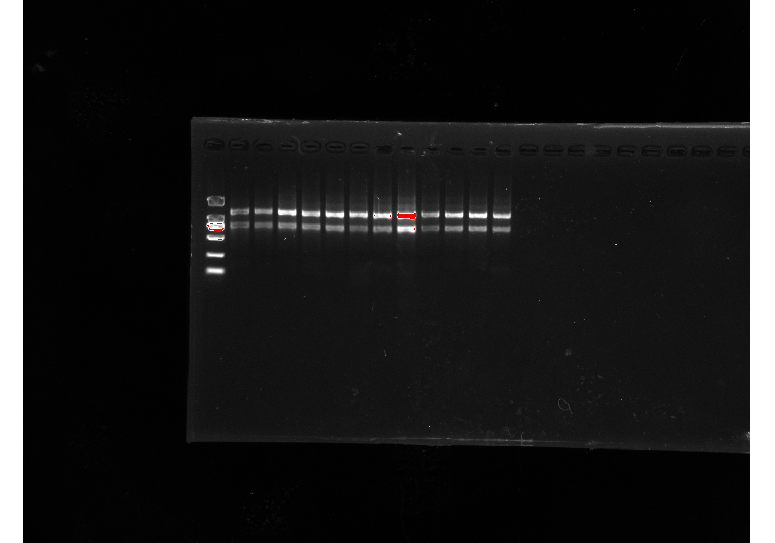


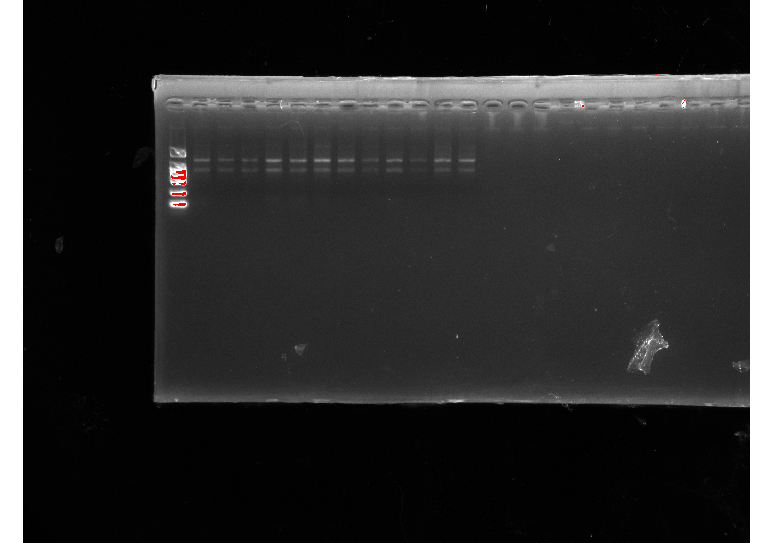


Supplementary file: **Quality control electrophoresis images of RNA extraction**

**Note**: Multiple RNA extractions were performed from Oncidium leaves during this study. The two electrophoretograms presented above show high-quality RNA samples from two successful extraction procedures. (Both gels were run with identical lane configurations as described below.)

Lanes 1 to 13 (from left to right):

**Lane 1:** Marker

**Lanes 2–7:** GR samples under heat stress at 0h, 1h, 2h, 4h, 8h, and 12h time points, respectively.

**Lanes 8–13:** HC samples under heat stress at 0h, 1h, 2h, 4h, 8h, and 12h time points, respectively.
