# Supplementary material for: Physiological and transcriptomic comparisons shed light on the high-temperature stress response mechanisms of Oncidium cultivars
Source: BMC Plant Biol. 2025 Sep 30;25:1242. doi: 10.1186/s12870-025-07254-7 (PMC12487527; doi:10.1186/s12870-025-07254-7)
Supplement: Supplementary file 4 — Supplementary Material 4. [file 12870_2025_7254_MOESM4_ESM.docx]

Supplementary Figures


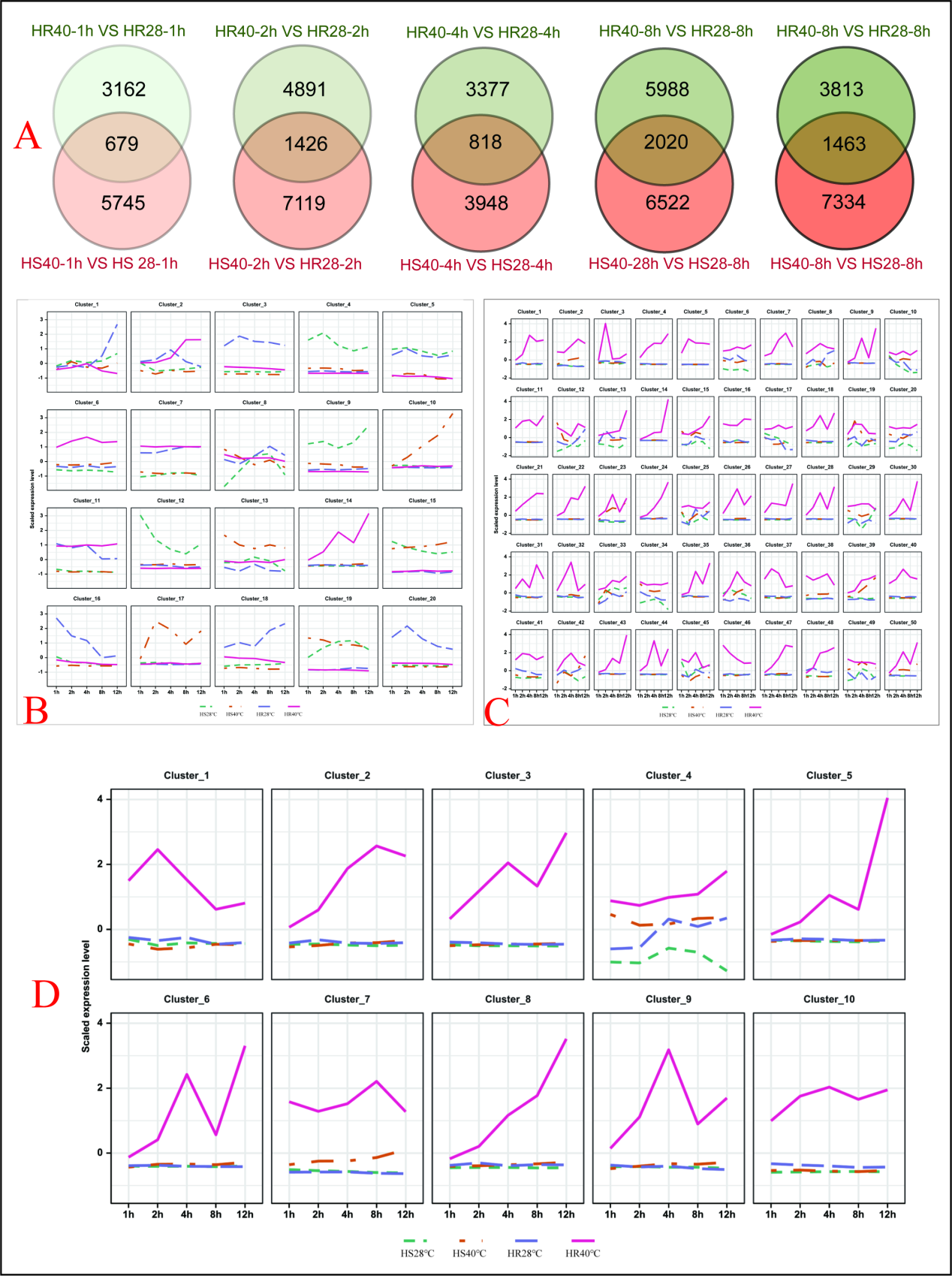


**Fig S1** Identification and functional analysis of heat-responsive genes in Oncidium cultivars under high-temperature stress.

**Notes:** (**A**) Volcano plot showing 26,683 differentially expressed genes (DEGs) identified between heat-tolerant (GR) and heat-sensitive (HC) cultivars at 40°C. (**B**) K-means clustering analysis of differentially expressed genes (DEGs) based on their FPKM expression profiles across time points, where the algorithm (k=20) grouped 26,683 DEGs into distinct clusters with similar expression patterns. (**C**)Secondary clustering analysis of 1,115 candidate genes from initial Clusters 6 and 14 using k-means algorithm (k=50) with identical normalization and distance metrics as primary analysis, where 12 non-responsive clusters (6,8,10,12,19,23,25,29,34,39,42,49) were excluded based on expression patterns, yielding 923 high-confidence heat-responsive genes for downstream analysis. (**D**) Final clustering analysis of 923 high-confidence heat-responsive genes.
