# Supplementary material for: Physiological and transcriptomic comparisons shed light on the high-temperature stress response mechanisms of Oncidium cultivars
Source: BMC Plant Biol. 2025 Sep 30;25:1242. doi: 10.1186/s12870-025-07254-7 (PMC12487527; doi:10.1186/s12870-025-07254-7)
Supplement: Supplementary file 5 — Supplementary Material 5. [file 12870_2025_7254_MOESM5_ESM.docx]

**Table S1** The primers used in the RT-qPCR assays.

| **Ggen Name** | **primer-F（5'-3')** | **primer-R（5'-3')** |
| --- | --- | --- |
| Actin | GGGCATATCCTTCGTAGATTGT | AATGTGCCTGCTATGTATGTTGCT |
| TRINITY_DN69689_c0_g1 | AAAACGACGGTAAGCACA | AGTAAGGGCGAGAAATGG |
| TRINITY_DN79246_c0_g2 | GCGGAAAGCATAATCCTAATC | TGAATCCCATACAACGAAACTG |
| TRINITY_DN80478_c3_g3 | CCATTTGCTCCACATACC | CATCTCCTTCACGGCTAC |
| TRINITY_DN76565_c0_g1 | TGGCGTGCTTACCGTCCTC | AACCCTCATCAACAGCAAAT |
| TRINITY_DN91276_c1_g4 | CCGCTATTGAACCCACTG | AGGGCACAACCATAAAGTAA |
| TRINITY_DN83818_c3_g2 | GGCTTACGCTTTTTTTGATTG | CGGTTGAGACGAGGGTTTC |
| TRINITY_DN76245_c5_g2 | ATCGCAACCAAAAACACATT | CAGGGCAACCACCTAAAAC |
| TRINITY_DN92890_c1_g5 | AATCAGCGGAGAGCGGAA | CCATAGCAGCCCTAACACC |
| TRINITY_DN97742_c3_g1 | GCTGCGGAATCCTGACTT | GCACTGGAGGTGGCAATCT |
| TRINITY_DN93930_c4_g6 | CTTTCGCCAACACACGCA | CGCCTTCAACCACCTCTACTT |
| TRINITY_DN85805_c1_g1 | CAGCCTCACCTTCTCCTCTAA | GGATGCGGATGCGTTGTA |
| TRINITY_DN64076_c0_g1 | GGAAGGAGAGGACAGAGCC | AATCACAGCAAACAGCCAAT |

**Table S2** Sample nomenclature and experimental design for heat-sensitive (HC) and heat-resistant (GR) *Oncidium* cultivars under control and high-temperature stress conditions

|  | **HC(heat-sensitive)** | |  | | **GR(heat- resistant)** | |  | |
| --- | --- | --- | --- | --- | --- | --- | --- | --- |
|  | **Control Group** |  | **Stress Group** |  | **Control Group** |  | **Stress Group** |  |
|  | HC280h_a |  |  |  | GR28H0_a |  |  |  |
|  | HC280h_b |  |  |  | GR28H0_b |  |  |  |
|  | HC280h_c |  |  |  | GR28H0_c |  |  |  |
|  | HC281h_a |  | HC401h_a |  | GR28H1_a |  | GR40H1_a |  |
|  | HC281h_b |  | HC401h_b |  | GR28H1_b |  | GR40H1_b |  |
|  | HC281h_c |  | HC401h_c |  | GR28H1_c |  | GR40H1_c |  |
|  | HC282h_a |  | HC402h_a |  | GR28H2_a |  | GR40H2_a |  |
|  | HC282h_b |  | HC402h_b |  | GR28H2_b |  | GR40H2_b |  |
|  | HC282h_c |  | HC402h_c |  | GR28H2_c |  | GR40H2_c |  |
|  | HC284h_a |  | HC404h_a |  | GR28H4_a |  | GR40H4_a |  |
|  | HC284h_b |  | HC404h_b |  | GR28H4_b |  | GR40H4_b |  |
|  | HC284h_c |  | HC404h_c |  | GR28H4_c |  | GR40H4_c |  |
|  | HC288h_a |  | HC408h_a |  | GR28H8_a |  | GR40H8_a |  |
|  | HC288h_b |  | HC408h_b |  | GR28H8_b |  | GR40H8_b |  |
|  | HC288h_c |  | HC408h_c |  | GR28H8_c |  | GR40H8_c |  |
|  | HC2812h_a |  | HC4012h_a |  | GR28H12_a |  | GR40H12_a |  |
|  | HC2812h_b |  | HC4012h_b |  | GR28H12_b |  | GR40H12_b |  |
|  | HC2812h_c |  | HC4012h_c |  | GR28H12_c |  | GR40H12_c |  |

**Notes**: ****HC****: Heat-sensitive cultivar; ****GR****: Heat-resistant cultivar Samples are labeled following the format: [Cultivar][Temperature°C][Durationh]_[Replicate] (e.g., HC281h_a = Heat-sensitive cultivar at 28°C, 1-hour treatment, replicate a) ****Control Group****: Maintained at 28°C; ****Stress Group****: Exposed to 40°C heat stress Biological triplicates (a, b, c) are provided for each condition, Duration (h) indicates hours of heat treatment (0h, 1h, 2h, 4h, 8h, 12h)

**Table S3** Summary of sequencing statistics for heat-sensitive (HC) and heat-resistant (GR) Oncidium sphacelatum cultivars under control (28°C) and high-temperature stress (40°C) conditions

| **Sample name** | **Raw reads** | **Raw bases** | **Clean reads** | **Clean bases** | **Error rate** | **Q20** | **Q30** | **GC content** |
| --- | --- | --- | --- | --- | --- | --- | --- | --- |
| HC280h_a | 45673272 | 6.88G | 45436244 | 6.86G | 0.03% | 96.98% | 92.06% | 46.84% |
| HC280h_b | 41007454 | 6.18G | 40737300 | 6.15G | 0.03% | 96.50% | 91.08% | 45.89% |
| HC280h_c |  |  |  |  |  |  |  |  |
| HC281h_a | 43697784 | 6.58G | 43425788 | 6.56G | 0.03% | 96.60% | 91.28% | 46.65% |
| HC281h_b | 41115564 | 6.19G | 40862968 | 6.17G | 0.03% | 96.73% | 91.59% | 45.95% |
| HC281h_c | 40885806 | 6.15G | 40582660 | 6.13G | 0.03% | 96.56% | 91.22% | 45.37% |
| HC282h_a | 42695188 | 6.43G | 42366512 | 6.4G | 0.03% | 96.70% | 91.57% | 44.96% |
| HC282h_b | 49581520 | 7.47G | 49326640 | 7.45G | 0.03% | 97.74% | 93.57% | 46.03% |
| HC282h_c | 40428714 | 6.09G | 40207282 | 6.07G | 0.03% | 97.11% | 92.36% | 45.50% |
| HC284h_a | 40767208 | 6.14G | 40579734 | 6.13G | 0.03% | 97.09% | 92.35% | 46.19% |
| HC284h_b | 47182220 | 7.11G | 46936230 | 7.09G | 0.03% | 97.12% | 92.40% | 45.75% |
| HC284h_c | 46841864 | 7.06G | 46639066 | 7.04G | 0.03% | 97.21% | 92.58% | 46.05% |
| HC288h_a | 45158394 | 6.8G | 44955090 | 6.79G | 0.03% | 97.33% | 92.85% | 46.51% |
| HC288h_b | 41202298 | 6.2G | 40995338 | 6.19G | 0.03% | 97.28% | 92.72% | 46.17% |
| HC288h_c | 45350236 | 6.83G | 45053440 | 6.8G | 0.03% | 97.01% | 92.20% | 46.12% |
| HC2812h_a | 46017104 | 6.93G | 45781330 | 6.91G | 0.03% | 97.08% | 92.30% | 46.23% |
| HC2812h_b | 44268808 | 6.66G | 44036496 | 6.65G | 0.03% | 97.39% | 92.97% | 46.53% |
| HC2812h_c | 40588430 | 6.11G | 40354522 | 6.09G | 0.03% | 97.02% | 92.21% | 45.76% |
| HC280h_a | 42642144 | 6.42G | 42416974 | 6.4G | 0.03% | 97.29% | 92.78% | 45.40% |
| HC401h_a | 43863066 | 6.61G | 43627842 | 6.59G | 0.03% | 97.05% | 92.31% | 47.32% |
| HC401h_b | 43250072 | 6.51G | 43012296 | 6.49G | 0.03% | 97.16% | 92.51% | 47.43% |
| HC401h_c | 43725856 | 6.59G | 43482846 | 6.57G | 0.03% | 97.09% | 92.39% | 47.62% |
| HC402h_a | 47692220 | 7.18G | 47442310 | 7.16G | 0.03% | 97.11% | 92.42% | 47.70% |
| HC402h_b | 49408484 | 7.44G | 49157686 | 7.42G | 0.03% | 97.18% | 92.54% | 47.48% |
| HC402h_c | 49669474 | 7.46G | 49260778 | 7.44G | 0.03% | 97.28% | 92.81% | 47.26% |
| HC404h_a | 38563642 | 5.8G | 38343602 | 5.79G | 0.03% | 97.19% | 92.60% | 47.51% |
| HC404h_b | 42394748 | 6.38G | 42108426 | 6.36G | 0.03% | 97.02% | 92.26% | 47.25% |
| HC404h_c | 38972004 | 5.86G | 38731520 | 5.85G | 0.03% | 97.08% | 92.38% | 47.24% |
| HC408h_a | 40962940 | 6.16G | 40720484 | 6.15G | 0.03% | 97.36% | 92.99% | 48.60% |
| HC408h_b | 43599302 | 6.56G | 43323970 | 6.54G | 0.03% | 97.10% | 92.44% | 47.62% |
| HC408h_c | 40965914 | 6.16G | 40673796 | 6.14G | 0.03% | 97.22% | 92.75% | 47.27% |
| HC4012h_a | 40818450 | 6.14G | 40575650 | 6.13G | 0.03% | 97.08% | 92.41% | 47.93% |
| HC4012h_b | 44370250 | 6.68G | 44077846 | 6.66G | 0.03% | 97.16% | 92.57% | 47.84% |
| HC4012h_c | 37751882 | 5.68G | 37492624 | 5.66G | 0.03% | 97.02% | 92.32% | 47.43% |
| GR28H0_a | 42256694 | 6.36G | 42016574 | 6.34G | 0.03% | 97.05% | 92.31% | 46.08% |
| GR28H0_b | 51464680 | 7.75G | 51175658 | 7.73G | 0.03% | 97.18% | 92.60% | 46.50% |
| GR28H0_c | 45780370 | 6.89G | 45509200 | 6.87G | 0.03% | 97.27% | 92.78% | 46.11% |
| GR28H1_a | 57128588 | 8.61G | 56834392 | 8.58G | 0.03% | 97.15% | 92.50% | 46.41% |
| GR28H1_b | 44977048 | 6.77G | 44740288 | 6.76G | 0.03% | 97.10% | 92.40% | 46.39% |
| GR28H1_c | 58190184 | 8.77G | 57864442 | 8.74G | 0.03% | 97.00% | 92.21% | 46.68% |
| GR28H2_a | 90304386 | 13.61G | 89875266 | 13.57G | 0.03% | 97.25% | 92.72% | 46.60% |
| GR28H2_b | 61355880 | 9.25G | 61058572 | 9.22G | 0.03% | 96.97% | 92.14% | 46.58% |
| GR28H2_c | 44293516 | 6.67G | 44039006 | 6.65G | 0.03% | 97.17% | 92.59% | 45.44% |
| GR28H4_a | 61681864 | 9.29G | 61365802 | 9.27G | 0.03% | 97.04% | 92.29% | 46.22% |
| GR28H4_b | 55841202 | 8.41G | 55515774 | 8.38G | 0.03% | 97.03% | 92.26% | 46.14% |
| GR28H4_c | 43002186 | 6.48G | 42784936 | 6.46G | 0.03% | 97.13% | 92.45% | 46.44% |
| GR28H8_a | 60599266 | 9.13G | 60287658 | 9.1G | 0.03% | 97.03% | 92.26% | 46.70% |
| GR28H8_b | 40871370 | 6.16G | 40659912 | 6.14G | 0.03% | 97.08% | 92.37% | 46.68% |
| GR28H8_c | 70283276 | 10.59G | 69896394 | 10.55G | 0.03% | 97.20% | 92.67% | 46.71% |
| GR28H12_a | 42010324 | 6.33G | 41837452 | 6.32G | 0.03% | 97.35% | 92.88% | 47.16% |
| GR28H12_b | 48049756 | 7.24G | 47796498 | 7.22G | 0.03% | 97.00% | 92.18% | 46.99% |
| GR28H12_c | 39247970 | 5.91G | 39031596 | 5.89G | 0.03% | 97.06% | 92.30% | 46.63% |
| GR40H1_a | 38960758 | 5.87G | 38804940 | 5.86G | 0.03% | 97.55% | 93.34% | 48.09% |
| GR40H1_b | 40546240 | 6.11G | 40335450 | 6.09G | 0.03% | 97.29% | 92.81% | 47.70% |
| GR40H1_c | 61147928 | 9.21G | 60857382 | 9.19G | 0.03% | 97.28% | 92.81% | 47.98% |
| GR40H2_a | 41197982 | 6.2G | 40972592 | 6.19G | 0.03% | 97.18% | 92.63% | 48.03% |
| GR40H2_b | 42774622 | 6.44G | 42566782 | 6.43G | 0.03% | 97.27% | 92.74% | 47.73% |
| GR40H2_c | 52794552 | 7.95G | 52513690 | 7.93G | 0.03% | 97.07% | 92.41% | 48.08% |
| GR40H4_a | 43962796 | 6.62G | 43712140 | 6.6G | 0.03% | 97.14% | 92.51% | 47.47% |
| GR40H4_b | 55839810 | 8.41G | 55519342 | 8.38G | 0.03% | 97.10% | 92.46% | 47.89% |
| GR40H4_c | 41174704 | 6.2G | 40960226 | 6.18G | 0.03% | 97.49% | 93.26% | 48.05% |
| GR40H8_a | 40187114 | 6.05G | 39943594 | 6.03G | 0.03% | 97.18% | 92.63% | 48.05% |
| GR40H8_b | 40607930 | 6.11G | 40356296 | 6.09G | 0.03% | 96.97% | 92.15% | 47.58% |
| GR40H8_c | 44283690 | 6.67G | 43992048 | 6.64G | 0.03% | 97.03% | 92.29% | 47.51% |
| GR40H12_a | 43450864 | 6.54G | 43241736 | 6.53G | 0.03% | 97.33% | 92.89% | 48.04% |
| GR40H12_b | 43392916 | 6.53G | 43140536 | 6.51G | 0.03% | 97.07% | 92.37% | 47.73% |
| GR40H12_c | 39881500 | 6.01G | 39655436 | 5.99G | 0.03% | 97.21% | 92.69% | 47.77% |

****Notes:**** ****Metrics****: Raw/Clean: Total/filtered reads; Q20/Q30: Bases with ≥99%/99.9% accuracy; Error rate: ≤0.03%; GC: 45-48% (expected range); ****Quality****: All samples met thresholds (Q20>96%, Q30>90%).

**Table S4** Annotation of significantly upregulated unigenes in Oncidium sphacelatum under high-temperature stress conditions

| **code** | **Up-regulated gene** | **Annotated protein** | **number of unigenes** | |
| --- | --- | --- | --- | --- |
| 1 | HSP20 | small class I heat shock protein (17.2-26.5 kDa ) | 36 |  |
| 2 | Photosynthesis,Chloroa_b-bind/LHCP/PSI/PsbW/PsbY | Photosystem I chlorophyll a/b-binding protein /Magnesium-protoporphyrin IX monomethyl ester/Photosystem I reaction center subunit /Oxygen-evolving enhancer protein /Photosystem II reaction center protein /hexaprenyl pyrophosphate synthase, mitochondrial /Protochlorophyllide reductase A, chloroplastic，NADPH-protochlorophyllide oxidoreductase A | 81 |  |
| 3 | IQ//BAG | BAG family molecular chaperone regulator | 3 |  |
| 4 | RuBisCO activase | Ribulose bisphosphate carboxylase/oxygenase activase | 4 |  |
| 5 | ubiquitin | Polyubiquitin | 17 |  |
| 6 | B_lectin/CVA | Mannose-specific lectin 1,Agglutinin,CVA-DOM1,CVA-DOM2 | 5 |  |
| 7 | ART3 | Antisense to ribosomal RNA transcript protein |  |  |
| 8 | LTP_2/Tryp_alpha_amyl//DUF2964 | Non-specific lipid-transfer protein | 4 |  |
| 9 | Mito_carr/PUMP6 | Mitochondrial uncoupling protein | 4 |  |
| 10 | Inhibitor_I29//Peptidase_C1/Pro-cathepsin H（BA） | Pro-cathepsin H,Cathepsin BA,Cathepsin H mini chain | 9 |  |
| 11 | Gp_dh_N | Glyceraldehyde-3-phosphate dehydrogenase | 2 |  |
| 12 | NAD-GH//HSP70//Radial_spoke_3 | Heat shock 70 kDa protein | 28 |  |
| 13 | Pro_CA | Beta carbonic anhydrase ,Beta carbonate dehydratase | 8 |  |
| 14 | TCTP | Translationally-controlled tumor protein homolog | 3 |  |
| 15 | BURP | BURP domain-containing protein BNM2A,Brassica napus microspore-derived embryo protein | 4 |  |
| 16 | HSP90//Met_synt_B12 | Heat shock protein 90 | 11 |  |
| 17 | Ribonuclease_T2 | Ribonuclease S-4 | 2 |  |
| 18 | HSF_DNA-bind | Heat stress transcription factor A-2a | 3 |  |
| 19 | ABA_WDS//MASE5 | Abscisic stress-ripening protein | 3 |  |
| 20 | BTB/zf-TAZ | BTB/POZ and TAZ domain-containing protein | 5 |  |
| 21 | peroxidase | L-ascorbate peroxidase 4, peroxisomal | 2 |  |
| 22 | RcbX | Chaperonin-like RbcX protein | 2 |  |
| 23 | MetOD1 | Uncharacterized mitochondrial protein |  |  |
| 24 | AAA_lid_9//AAA_2 | Chaperone protein ClpB | 5 |  |
| 25 | Catalase//Catalase-rel | Catalase isozyme | 5 |  |
| 26 | TIM | Triosephosphate isomerase,Triose-phosphate isomerase |  |  |
| 27 | Glycolytic//DUF647/FBA | Fructose-bisphosphate aldolase | 7 |  |
| 28 | NAF//Trimer_CC/Pkinase/PKS | CBL-interacting serine/threonine-protein kinase,SNF1-related kinase,SOS2-like protein kinase PKS | 6 |  |
| 29 | Auxin_inducible | Auxin-responsive protein SAUR41[Arabidopsis thaliana] |  |  |
| 30 | Asp//SapB_2//SapB_1 | Procardosin-A, Cardosin-A intermediate form 35 kDa subunit | 4 |  |
| 31 | Lipocalin_2 | Apolipoprotein D |  |  |
| 32 | Thi4 | Thiamine thiazole synthase, chloroplastic,Thiazole biosynthetic enzyme [Arabidopsis thaliana] | 3 |  |
| 33 | FKBP_C | Peptidyl-prolyl cis-trans isomerase FKBP5 | 3 |  |
| 34 | Glyoxalase | Lactoylglutathione lyase，Aldoketomutase，Glyoxalase I，Ketone-aldehyde mutase， Methylglyoxalase [ | 2 |  |
| 35 | rve | Pro-Pol polyprotein, Protease/Reverse transcriptase/ribonuclease H | 2 |  |
| 36 | IN, CA, NC, RT | Transposon Ty3-G Gag-Pol polyprotein, Transposon Ty3-1 TYA-TYB polyprotein, Reverse transcriptase/ribonuclease H | 3 |  |
| 37 | HMA | Copper transport protein ATOX1, Metal transport protein ATX1 |  |  |
| 38 | iPGM_N//Metalloenzyme | 2,3-bisphosphoglycerate-independent phosphoglycerate mutase，Phosphoglyceromutase | 6 |  |
| 39 | NifU_N | Iron sulfur cluster assembly protein 1, mitochondrial |  |  |
| 40 | ADH_N//ADH_zinc_N | Zinc-type alcohol dehydrogenase-like protein C16A3.02c | 3 |  |
| 41 | DUF5485//Glyco_hydro_16//XET_C | xyloglucan endotransglucosylase/hydrolase protein | 5 |  |
| 42 | Bax1-I | Testis-enhanced gene transcript protein，Transmembrane BAX inhibitor motif-containing protein 6 | 1 |  |
| 43 | VPE1 | Vacuolar-processing enzyme beta-isozyme 1 | 2 |  |
| 44 | Thioredoxin | Thioredoxin H-type 2 |  |  |
| 45 | DnaJ//RPT//AbLIM_anchor | DnaJ homolog subfamily B member 6-A | 6 |  |
| 46 | FAE1_CUT1_RppA | 3-ketoacyl-CoA synthase 11，Very long-chain fatty acid condensing enzyme 11，VLCFA condensing enzyme 11 | 1 |  |
| 47 | Cpn60_TCP1 | 60 kDa chaperonin 2，roEL protein ，Protein Cpn60 2 | 3 |  |
| 48 | NRP1 | Nodulin-related protein 1，RPS2-interacting protein 11 | 2 |  |
| 49 | Lir1 | Light-regulated protein | 2 |  |
| 50 | Histone | Histone H2B.4 | 2 |  |
| 51 | CBSX3 | CBS domain-containing protein CBSX3, mitochondrial | 1 |  |
| 52 | RVT_1 | enzymatic polyprotein，Protease，Reverse transcriptase，Ribonuclease H | 1 |  |
| 53 | GST_N//GST_C_2 | Glutathione S-transferase U23 [Arabidopsis thaliana]，Glutathione peroxidase 1 | 4 |  |
| 54 | Phosphoesterase | Phospholipase | 2 |  |
| 55 | Cytochrom_B561 | Cytochrome b-c1 complex subunit 7，Complex III subunit VII，Ubiquinol-cytochrome c reductase complex 14 kDa protein | 3 |  |
| 56 | DIOX_N | Gibberellin 20 oxidase 1-D | 1 |  |
| 57 | Ribosomal_L14 | ribosomal protein | 9 |  |
| 58 | PMEI | Pectinesterase inhibitor 3，Pectin methylesterase inhibitor 3 | 2 |  |
| 59 | Na_Ca_ex | vacuolar cation/proton exchanger 6，Ca(2+)/H(+) exchanger 6 | 2 |  |
| 60 | Amidohydro_1 | Allantoin-utilizing enzyme | 2 |  |
| 61 | eIF-5A-2 | Eukaryotic translation initiation factor 5A-2 | 1 |  |
| 62 | Enolase_N//Enolase_C | 2-phosphoglycerate dehydratase | 1 |  |
| 63 | FMN_dh | RPeroxisomal (S)-2-hydroxy-acid oxidase GLO4，Glycolate oxidase 4 | 1 |  |
| 64 | KNOX2//ClpB_D2-small | Heat shock protein 100，Protein CLP | 1 |  |
| 65 | MIP//DUF1275 | aquaporin PIP2-6，Plasma membrane intrinsic protein 2-6 | 3 |  |
| 66 | Auxin_repressed | Dormancy-associated protein 1，Dormancy-associated protein-like | 1 |  |
| 67 | RicinB_lectin_2 | Ricin B-like lectin EULS3 | 1 |  |
| 68 | Ferric_reduct//FAD_binding_8//NAD_binding_6 | Respiratory burst oxidase homolog protein B，NADPH oxidase RBOHB | 1 |  |
| 69 | Str_synth | Protein STRICTOSIDINE SYNTHASE-LIKE 12 | 1 |  |
| 70 | EF1_GNE//p12I | Elongation factor 1-beta | 3 |  |
| 71 | BIC2 | Protein BIC2，BLUE-LIGHT INHIBITOR OF CRYPTOCHROMES 2 | 1 |  |
| 72 | FA_hydroxylase | Methylsterol monooxygenase，C-4 methylsterol oxidase，Sterol-C4-methyl oxidase | 1 |  |
| 73 | Mob1_phocein | MOB kinase activator-like 1 homolog C，Mps one binder kinase activator-like 1 homolog C | 1 |  |
| 74 | Bet_v_1 | Major pollen allergen Bet v 1-A | 1 |  |
| 75 | ORMDL | ORM1-like protein 2，Adoplin-2 | 1 |  |
| 76 | ARD | 1,2-dihydroxy-3-keto-5-methylthiopentene dioxygenase 3，Acireductone dioxygenase (Fe(2+)-requiring) | 1 |  |
| 77 | Arf | ADP-ribosylation factor 1 | 1 |  |
| 78 | TAL_FSA | Transaldolase 2 | 1 |  |
| 79 | Ldh_1_N//Ldh_1_C | Malate dehydrogenase | 1 |  |
| 80 | PP2C//DUF4440//DUF4181 | protein phosphatase | 1 |  |
| 81 | LacAB_rpiB | Galactose-6-phosphate isomerase subunit LacB 2 | 1 |  |
| 82 | Amino_oxidase | Polyamine oxidase 4 | 1 |  |
| 83 | Glutaredoxin | Glutaredoxin | 1 |  |
| 84 | 2OG-FeII_Oxy | RecFlavonol synthase/flavanone 3-hydroxylase | 1 |  |
| 85 | UDP-glycosyltransferase | UDP-glycosyltransferase 85A4 | 1 |  |
| 86 | RRM_1 | Polyadenylate-binding protein RBP47B'，Poly(A)-binding protein RBP47B' | 1 |  |
| 87 | zf-FLZ | FCS-Like Zinc finger 7 | 1 |  |
| 88 | zf-AN1 | AN1-type zinc finger protein 2B，Arsenite-inducible RNA-associated protein-like protein | 1 |  |
| 89 | RT_RNaseH_2 | Gag-Pol polyprotein，Matrix protein p15，RNA-binding phosphoprotein p12，Capsid protein p30，Nucleocapsid protein p10-Pol，Protease，Reverse transcriptase/ribonuclease HN | 1 |  |
| 90 | RVT_1 | Transposon Tf2-11 polyprotein，Retrotransposable element Tf2 155 kDa protein | 1 |  |
| 91 | 2-Hacid_dh//2-Hacid_dh_C | 2-ketogluconate reductase | 1 |  |
|  |  |  |  |  |

****Notes:** The table presents significantly upregulated unigenes with: (1) serial numbers in Column 1, (2) gene categories in Column 2, (3) protein functions predicted by homology in Column 3, and (4) transcript counts per category in Column 4. Major upregulated pathways include heat shock proteins (HSP20, HSP70, HSP90), photosynthesis-related proteins, and stress-responsive factors (BAG family, ubiquitin pathway). Multiple chaperones (e.g., ClpB, Cpn60) and redox regulators (e.g., peroxidases, thioredoxins) were significantly induced. All annotations were obtained through BLASTx searches (E-value < 1e-5 against Nr/UniProt databases), with functional grouping based on GO and KEGG enrichment (FDR < 0.05).**

**Table S5** Functional annotation of significantly downregulated unigenes in Oncidium sphacelatum under high-temperature stress

| **code** | **Down-regulated gene** | **Annotated protein** | **number of unigenes** | |
| --- | --- | --- | --- | --- |
| 1 | HATPase_c//ubiquitin | Polyubiquitin | 6 |  |
| 2 | RbcS//RuBisCO_small//PPE/RPE | Ribulose bisphosphate carboxylase small chain SSU5A, RuBisCO small subunit SSU5A | 7 |  |
| 3 | GDC-P/Glycine dehydrogenase | Glycine dehydrogenase (decarboxylating) | 6 |  |
| 4 | PsaL//PsbR//CobN-Mg_chel | Photosystem I reaction center subunit XI[Synechocystis sp. PCC 6803 substr. Kazusa]Photosystem II protein PSBR，Protein PHOTOSYSTEM II SUBUNIT R | 3 |  |
| 5 | hydrogenase | Soluble hydrogenase 42 kDa subunit | 1 |  |
| 6 | PEPcase | Phosphoenolpyruvate carboxylase | 4 |  |
| 7 | Metallothio_2 | Metallothionein-like protein 4A，Class I metallothionein-like protein 4A | 1 |  |
| 8 | DUF3349//EF-hand_7//DUF4220//AGP | Calmodulin | 1 |  |
| 9 | Gp_dh_N/Gp_dh_C | Glyceraldehyde-3-phosphate dehydrogenase B，NADP-dependent glyceraldehydephosphate dehydrogenase subunit B | 7 |  |
| 10 | H_PPase | K(+)-insensitive pyrophosphate-energized proton pump，Membrane-bound proton-translocating pyrophosphatase，Pyrophosphate-energized inorganic pyrophosphatase | 5 |  |
| 11 | Pkinase// | Calcium-dependent protein kinase 11 | 1 |  |
| 12 | SGAT/AGT | Serine--glyoxylate aminotransferase，Alanine--glyoxylate aminotransferase | 1 |  |
| 13 | Copper-bind | Plastocyanin | 1 |  |
| 14 | Gln-synt_N//Gln-synt_C//DUF3953 | Glutamine synthetase cytosolic isozyme 2，Glutamate--ammonia ligase | 1 |  |
| 15 | Aminotran_5 | (S)-ureidoglycine--glyoxylate transaminase，Purine catabolism protein PucG | 2 |  |
| 16 | Ldh_1_N//Ldh_1_C | Malate dehydrogenase，Cytosolic malate dehydrogenase | 3 |  |
| 17 | AAA_lid_9//AAA_2//ClpB_D2-small | ATP-dependent Clp protease ATP-binding subunit ClpA homolog | 10 |  |
| 18 | MSP | Oxygen-evolving enhancer protein 1, chloroplastic，33 kDa subunit of oxygen evolving system of photosystem II，33 kDa thylakoid membrane protein; AltName | 3 |  |
| 19 | DEAD//Helicase_C//YgbB | DEAD-box ATP-dependent RNA helicase 7 | 7 |  |
| 20 | SHMT | Serine hydroxymethyltransferase，Glycine hydroxymethyltransferase，Glycosylation-related protein 1，Maternal effect lethal protein 32，Serine methylase | 3 |  |
| 21 | GATase_2//Glu_syn_central//Glu_synthase//GXGXG | Ferredoxin-A, Ferredoxin-dependent glutamate synthase | 4 |  |
| 22 | B_lectin//ZZ | 24 kDa actin-binding protein，CABP1-related protein p24 | 1 |  |
| 23 | zf-RVT//RVT_3 | Putative ribonuclease H protein | 1 |  |
| 24 | LRR_8 | leucine-rich repeat receptor-like protein kinase | 5 |  |
| 25 | GTP_EFTU//IF-2//GTP_EFTU_D2 | Translation initiation factor IF-2 | 2 |  |
| 26 | Cytochrom_C_asm//PetM | Cytochrome b6-f complex subunit 7，Cytochrome b6-f complex subunit PetM | 2 |  |
| 27 | Cupin_1 | Germin-like protein | 1 |  |
| 28 | PPDK_N | phosphoenolpyruvate synthasePyruvate, water dikinase | 2 |  |
| 29 | EF_TS | Elongation factor Ts | 7 |  |
| 30 | methyltransferase | Isoflavone 7-O-methyltransferase，Daidzein 7-O-methyltransferase | 1 |  |
| 31 | S-AdoMet_synt_N//S-AdoMet_synt_M | S-adenosylmethionine synthase，Methionine adenosyltransferase | 3 |  |
| 32 | zf-FLZ | FCS-Like Zinc finger 13 | 2 |  |
| 33 | Catalase | Catalase isozyme C | 3 |  |
| 34 | Phosphorylase//FAM104//DUF4982//GP1 | Glycogen phosphorylase 1 | 2 |  |
| 35 | MPBQ/MSBQ methyltransferase/E37 | 2-methyl-6-phytyl-1,4-hydroquinone methyltransferase，7 kDa inner envelope membrane protein，MPBQ/MSBQ methyltransferase; ，Protein ALBINO OR PALE GREEN MUTANT 1 | 1 |  |
| 36 | Mito_carr | Mitochondrial uncoupling protein 2 | 2 |  |
| 37 | DUF4922 | GDP-D-glucose phosphorylase 1 | 2 |  |
| 38 | RRM_1 | 29 kDa ribonucleoprotein, chloroplastic，RNA-binding protein CP29A | 6 |  |
| 39 | Sucrose_synth//Glycos_transf_1 | Sucrose synthase，Nodulin-100，Sucrose-UDP glucosyltransferase | 2 |  |
| 40 | pNd3/pNd10 | Disease resistance protein RPS5，Resistance to Pseudomonas syringae protein 5 | 2 |  |
| 41 | NAM | NAC domain-containing protein 2 | 1 |  |
| 42 | ArgoMid//Piwi | Protein argonaute 14 | 5 |  |
| 43 | Frigida//FRL2 | Inactive FRIGIDA-like protein 2 | 1 |  |
| 44 | ACP_syn_III_C//FAE1_CUT1_RppA | 3-ketoacyl-CoA synthase 11，Very long-chain fatty acid condensing enzyme 11，VLCFA condensing enzyme 11 | 8 |  |
| 45 | Glycolytic//SMYLE_N | Fructose-bisphosphate aldolase, cytoplasmic isozyme 2 | 1 |  |
| 46 | Rx_N//NB-ARC | Putative disease resistance protein RGA4 | 1 |  |
| 47 | NB-ARC | disease resistance protein RXW24L | 7 |  |
| 48 | Rhodanese | Rhodanese-like domain-containing protein 9，Sulfurtransferase 9 | 1 |  |
| 49 | NAD-GH//HSP70 | Chaperone protein dnaK2，Heat shock 70 kDa protein | 2 |  |
| 50 | Cpn60_TCP1 | Protein Cpn60 1 | 1 |  |
| 51 | ECT4/YTH | YTH domain-containing protein ECT4，Protein EVOLUTIONARILY CONSERVED C-TERMINAL REGION 4 | 3 |  |
| 52 | Polyketide_cyc2 | Abscisic acid receptor PYL11，Regulatory components of ABA receptor 5 | 1 |  |
| 53 | Acyl-thio_N | Acyl-[acyl-carrier-protein] hydrolase FATB3，Myristoyl-[acyl-carrier-protein] thioesterase | 1 |  |
| 54 | Terpene_synth//LdTPS6 | Beta-caryophyllene synthase,Terpene synthase | 2 |  |
| 55 | DUF4220//AGP | Arabinogalactan protein 22 | 3 |  |
| 56 | AhpC-TSA | Putative peroxiredoxin MT2597，Bacterioferritin comigratory protein，Thioredoxin peroxidase，hioredoxin-dependent peroxiredoxin MT2597 [Mycobacterium tuberculosis CDC1551] | 1 |  |
| 57 | MIP | aquaporin TIP4-3，Tonoplast intrinsic protein 4-3 | 2 |  |
| 58 | S1 | Transcriptional accessory protein Tex | 1 |  |
| 59 | CP12 | Calvin cycle protein CP12，CP12 domain-containing protein | 1 |  |
| 60 | MRS2-B | Magnesium transporter MRS2-B | 1 |  |
| 61 | Actin | Actin-97 | 3 |  |
| 62 | UTP15_C//Acyltransferase | glycerol-3-phosphate acyltransferase 2 | 1 |  |
| 63 | Ribosomal_S10 | 40S ribosomal protein S20 | 3 |  |
| 64 | AMY3 | Alpha-amylase 3，1,4-alpha-D-glucan glucanohydrolase | 1 |  |
| 65 | F-box | F-box/kelch-repeat protein | 1 |  |
| 66 | Dimerisation//Methyltransf_2 | (Iso)eugenol O-methyltransferase EOMT1，S-adenosysl-L-methionine | 1 |  |
| 67 | Epimerase | Chloroplast stem-loop binding protein of 41 kDa a | 1 |  |
| 68 | fn3_6 | Subtilisin-like protease SBT3 | 1 |  |
| 69 | 1,2 rhamnosyltransferase | Flavanone 7-O-glucoside 2''-O-beta-L-rhamnosyltransferase | 1 |  |
| 70 | TAXi_N | Protein ASPARTIC PROTEASE IN GUARD CELL 1 | 2 |  |
| 71 | NPH3 | BTB/POZ domain-containing protein | 1 |  |
| 72 | CobN-Mg_chel | Aerobic cobaltochelatase subunit CobN，Hydrogenobyrinic acid a,c-diamide cobaltochelatase subunit CobN | 5 |  |
| 73 | Histone | Histone H2B.4 |  |  |
| 74 | LTP_2 | Putative lipid-transfer protein DIR1 | 2 |  |
| 75 | Tubulin | Tubulin beta-2 chain | 3 |  |
| 76 | zf-UDP//FYVE_2//Cellulose_synt | cellulose synthase A catalytic subunit 2 | 2 |  |
| 77 | Aldedh | Aldehyde dehydrogenase | 1 |  |
| 78 | SMXL2 | Protein SMAX1-LIKE 2 | 1 |  |
| 79 | Meth_synt_2 | 5-methyltetrahydropteroyltriglutamate--homocysteine methyltransferas，Cobalamin-independent methionine synthase，Methionine synthase, vitamin-B12 independent isozyme | 5 |  |
| 80 | peroxidase | Peroxidase 15 | 1 |  |
| 81 | AdoHcyase//AdoHcyase_NAD | Adenosylhomocysteinase，S-adenosyl-L-homocysteine hydrolase | 1 |  |
| 82 | Bet_v_1 | Ribonuclease 1 | 1 |  |
| 83 | Topoisom_I//PspA_IM30 | DNA topoisomerase 1 | 1 |  |
| 84 | - | Uncharacterized protein | 1 |  |
| 85 | - | Protein PLASTID MOVEMENT IMPAIRED 1 | 1 |  |
| 86 | COPI_C//zinc_ribbon_9 | Coatomer subunit alpha，Alpha-coat protein，Xenopsin-related peptide | 3 |  |
| 87 | LEAP-2//S1 | Polyprotein of EF-Ts，Plastid-specific ribosomal protein-7，Elongation factor Ts | 1 |  |
| 88 | AUX_IAA | Auxin response factor | 1 |  |
| 89 | HisKA//HATPase_c | Phytochrome A | 1 |  |
| 90 | Pro_isomerase | Peptidyl-prolyl cis-trans isomerase B | 1 |  |
| 91 | Ras | GTP-binding nuclear protein Ran-2，Ras-related nuclear protein | 1 |  |
| 92 | Glucan_synthase | Callose synthase 9，Protein GLUCAN SYNTHASE-LIKE 10 | 1 |  |
| 93 | tify//CCT_2 | Protein TIFY 11d | 1 |  |
| 94 | TSS | Protein TSS，TPR-domain suppressor of STIMPY | 1 |  |
| 95 | FYVE_2//zf-B_box | Zinc finger protein CONSTANS-LIKE 7 | 1 |  |
| 96 | UGT1 | Crocetin glucosyltransferase, chloroplastic，UDP-glucose glucosyltransferase 1，UDP-glycosyltransferase | 1 |  |
| 97 | adh_short//WGG | RTropinone reductase 2 | 1 |  |
| 98 | Myb_DNA-binding//Peptidase_M7 | Myb-like protein G | 1 |  |
| 99 | DUF1156//LRR_8//Pkinase_Tyr | Leucine-rich repeat receptor-like serine/threonine-protein kinase | 1 |  |
| 100 | ThylakoidFormat | Protein THYLAKOID FORMATION1, chloroplastic | 1 |  |
| 101 | PDGLE | receptor-like protein kinase | 1 |  |
| 102 | CRAL_TRIO_N//CRAL_TRIO | SEC14 cytosolic factor，Phosphatidylinositol/phosphatidylcholine transfer protein | 1 |  |
| 103 | ANTH | Clathrin coat assembly protein AP180，Clathrin coat-associated protein | 1 |  |
| 104 | ABC_tran | ABC transporter G family member | 1 |  |
| 105 | BTB | Coleoptile phototropism protein 1，BTB/POZ domain-containing protein CPT1，Non-phototropic hypocotyl 3-like protein | 1 |  |
| 106 | ADC2 | Arginine decarboxylase 2 | 1 |  |
| 107 | Lipoxygenase | Linoleate 9S-lipoxygenase 1 | 1 |  |
| 108 | Sod_Cu | Superoxide dismutase [Cu-Zn], chloroplastic | 1 |  |

****Notes:** The table presents significantly downregulated unigenes with: (1) serial numbers in Column 1, (2) gene categories in Column 2, (3) protein functions predicted by homology in Column 3, and (4) transcript counts per category in Column 4. Major downregulated pathways include photosynthesis components (RuBisCO, photosystem subunits), energy metabolism (GAPDH, malate dehydrogenase), and stress responses (metallothioneins, peroxidases). Uncharacterized proteins are marked with "-". All annotations were obtained through BLASTx searches (E-value<1e-5 against NCBI Nr database), with significant downregulation defined as ≥2-fold decrease (FDR-adjusted p<0.05).**
