# Supplementary material for: Physiological and transcriptomic comparisons shed light on the high-temperature stress response mechanisms of Oncidium cultivars
Source: BMC Plant Biol. 2025 Sep 30;25:1242. doi: 10.1186/s12870-025-07254-7 (PMC12487527; doi:10.1186/s12870-025-07254-7)
Supplement: Supplementary file 3 — Supplementary Material 3. [file 12870_2025_7254_MOESM3_ESM.zip › 8.DiffExprAnalysis/8.1.DEGsList/DEG_statistics.pdf]

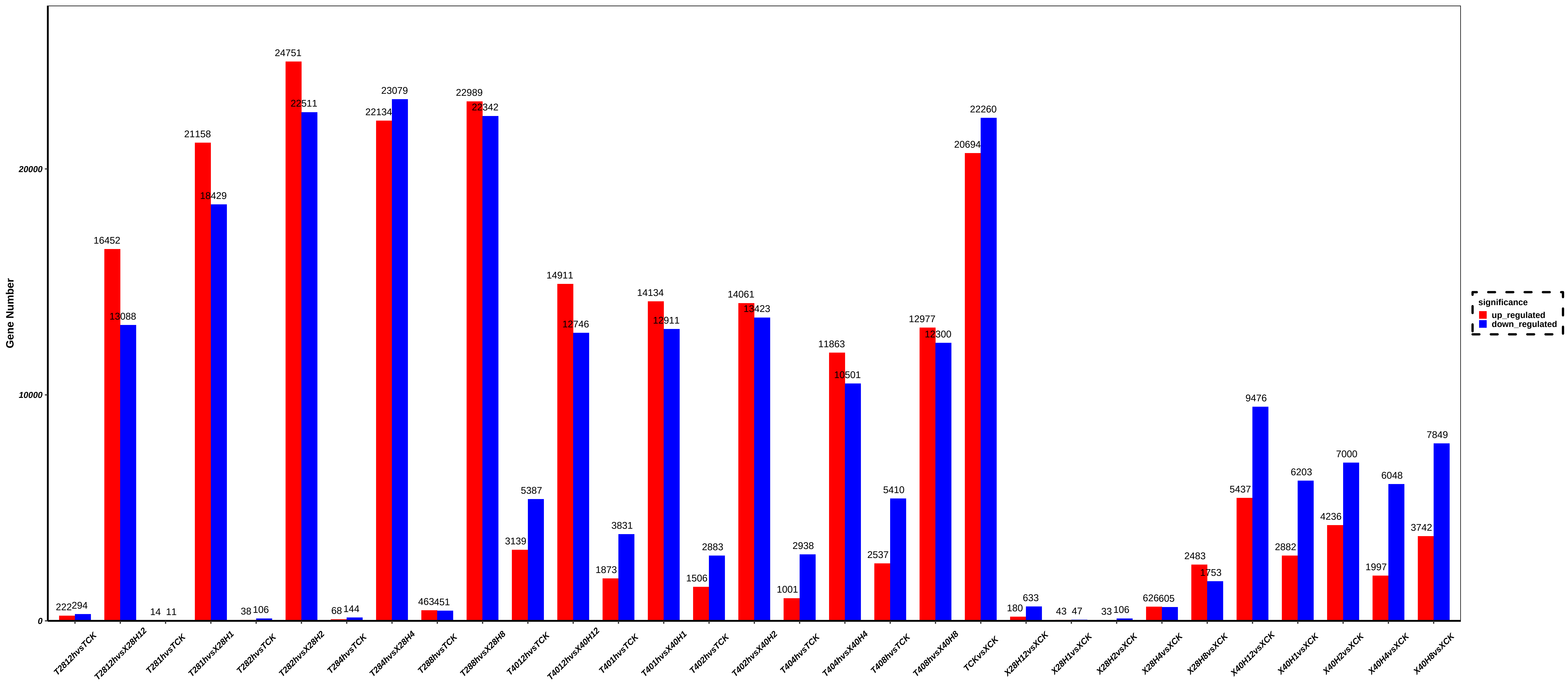

Gene Number
20000
10000
0
significance
up_regulated
down_regulated
222
294
16452
13088
14
11
21158
18429
38
106
24751
22511
68
144
22134
23079
463
451
22989
22342
3139
5387
14911
12746
1873
3831
14134
12911
1506
2883
14061
13423
1001
2938
11863
10501
2537
5410
12977
12300
20694
22260
180
633
43
47
33
106
626
605
2483
1753
5437
9476
2882
6203
4236
7000
1997
6048
3742
7849
T2812hvsTCK
T2812hvsX28H12
T281hvsTCK
T281hvsX28H1
T282hvsTCK
T282hvsX28H2
T284hvsTCK
T284hvsX28H4
T288hvsTCK
T288hvsX28H8
T4012hvsTCK
T4012hvsX40H12
T401hvsTCK
T401hvsX40H1
T402hvsTCK
T402hvsX40H2
T404hvsTCK
T404hvsX40H4
T408hvsTCK
T408hvsX40H8
TCKvsXCK
X28H12vsXCK
X28H1vsXCK
X28H2vsXCK
X28H4vsXCK
X28H8vsXCK
X40H12vsXCK
X40H1vsXCK
X40H2vsXCK
X40H4vsXCK
X40H8vsXCK
