# Supplementary material for: Physiological and transcriptomic comparisons shed light on the high-temperature stress response mechanisms of Oncidium cultivars
Source: BMC Plant Biol. 2025 Sep 30;25:1242. doi: 10.1186/s12870-025-07254-7 (PMC12487527; doi:10.1186/s12870-025-07254-7)
Supplement: Supplementary file 3 — Supplementary Material 3. [file 12870_2025_7254_MOESM3_ESM.zip › 8.DiffExprAnalysis/8.1.DEGsList/T2812hvsTCK/T2812hvsTCK.volcano.pdf]

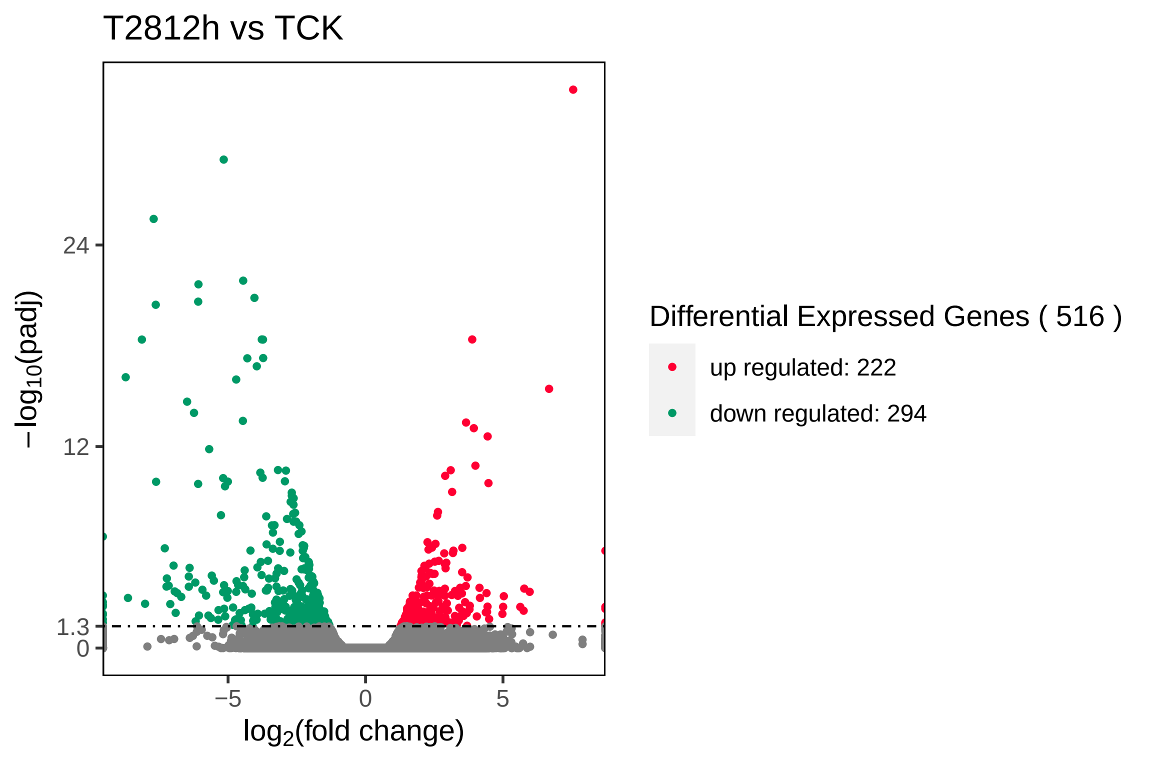
T2812h vs TCK
−log10(padj)
24
12
1.3
0
−5
0
5
log2(fold change)
Differential Expressed Genes (516)
up regulated: 222
down regulated: 294
